# Microenvironment and tumor inflammatory features improve prognostic prediction in gastro‐entero‐pancreatic neuroendocrine neoplasms

**DOI:** 10.1002/cjp2.135

**Published:** 2019-07-09

**Authors:** Massimo Milione, Rosalba Miceli, Francesco Barretta, Alessio Pellegrinelli, Paola Spaggiari, Giovanna Tagliabue, Giovanni Centonze, Cinzia Paolino, Alessandro Mangogna, Ketevani Kankava, Sara Pusceddu, Luca Giacomelli, Ambra Corti, Christian Cotsoglou, Vincenzo Mazzaferro, Gabriella Sozzi, Filippo de Braud, Giancarlo Pruneri, Andrea Anichini

**Affiliations:** ^1^ Department of Pathology and Laboratory Medicine Fondazione IRCCS – Istituto Nazionale dei Tumori Milan Italy; ^2^ Medical Statistics, Biometry and Bioinformatics, Unit of Clinical Epidemiology and Trial Organization Fondazione IRCCS – Istituto Nazionale dei Tumori Milan Italy; ^3^ Department of Pathology ASST Franciacorta, Mellino Mellini Hospital Chiari, Brescia Italy; ^4^ Department of Pathology Cancer Center Humanitas Research Hospital Milan Italy; ^5^ Cancer Registry Unit, Department of Preventive and Predictive Medicine Fondazione IRCCS – Istituto Nazionale dei Tumori Milan Italy; ^6^ Department of Research Fondazione IRCCS – Istituto Nazionale dei Tumori Milan Italy; ^7^ Unit of Pathology, Clinical Department of Medical Surgical and Health Science, University of Trieste, Ospedale di Cattinara Trieste Italy; ^8^ Teaching, Scientific and Diagnostic Pathology Laboratory Tbilisi State Medical University Tbilisi Georgia; ^9^ Medical Oncology Department Fondazione IRCCS – Istituto Nazionale dei Tumori Milan Italy; ^10^ Department of Surgical Sciences and Integrated Diagnostics University of Genoa Genoa Italy; ^11^ Polistudium SRL Milan Italy; ^12^ Hepato‐Bilio‐Pancreatic Surgery and Liver Transplantation Fondazione IRCCS – Istituto Nazionale dei Tumori Milan Italy; ^13^ School of Medicine University of Milan Milan Italy

**Keywords:** gastro‐entero‐pancreatic neuroendocrine neoplasm, microenvironment, immune and inflammatory markers, Ki‐67, morphology, disease‐free survival

## Abstract

Microenvironment‐related immune and inflammatory markers, when combined with established Ki‐67 and morphology parameters, can improve prognostic prediction in gastro‐entero‐pancreatic neuroendocrine neoplasms (GEP‐NENs). Therefore, we evaluated the prognostic value of microenvironment and tumor inflammatory features (MoTIFs) in GEP‐NENs. For this purpose, formalin‐fixed paraffin‐embedded tissue sections from 350 patients were profiled by immunohistochemistry for immune, inflammatory, angiogenesis, proliferation, NEN‐, and fibroblast‐related markers. A total of 314 patients were used to generate overall survival (OS) and disease‐free survival (DFS) MoTIFs prognostic indices (PIs). PIs and additional variables were assessed using Cox models to generate nomograms for predicting 5‐year OS and DFS. A total of 36 patients were used for external validation of PIs and nomograms' prognostic segregations. From our analysis, G1/G2 versus G3 GEP‐NENs showed phenotypic divergence with immune‐inflammatory markers. HLA, CD3, CD8, and PD‐1/PD‐L1 IHC expression separated G3 into two sub‐categories with high versus low adaptive immunity‐related features. MoTIFs PI for OS based on COX‐2^Tumor(T)^ > 4, PD‐1^Stromal(S)^ > 0, CD8^S^ < 1, and HLA‐I^S^ < 1 was associated with worst survival (hazard ratio [HR] 2.50; 95% confidence interval [CI], 2.12–2.96; *p* < 0.0001). MoTIFs PI for DFS was based on COX‐2^T^ > 4, PD‐1^S^ > 4, HLA‐I^S^ < 1, HLA‐I^T^ < 2, HLA‐DR^S^ < 6 (HR 1.77; 95% CI, 1.58–1.99; *p* < 0.0001). Two nomograms were developed including morphology (HR 4.83; 95% CI, 2.30–10.15; *p* < 0.001) and Ki‐67 (HR 11.32; 95% CI, 5.28–24.24; *p* < 0.001) for OS, and morphology (PI = 0: HR 10.23; 95% CI, 5.67–18.47; PI = 5: HR 2.87; 95% CI, 1.21–6.81; *p* < 0.001) and MoTIFs PI for DFS in well‐differentiated GEP‐NENs (HR 6.21; 95% CI, 2.52–13.31; *p* < 0.001). We conclude that G1/G2 to G3 transition is associated with immune‐inflammatory profile changes; in fact, MoTIFs combined with morphology and Ki‐67 improve 5‐year DFS prediction in GEP‐NENs. The immune context of a subset of G3 poorly differentiated tumors is consistent with activation of adaptive immunity, suggesting a potential for responsiveness to immunotherapy targeting immune checkpoints.

## Introduction

Gastro‐entero‐pancreatic neuroendocrine neoplasms (GEP‐NENs) are the most frequent neuroendocrine tumors (NETs) 1. The World Health Organization (WHO) 2010 classification divides GEP‐NENs into G1, G2, and G3, according to Ki‐67 and/or mitotic index (MI) 2. However, this classification was challenged by several studies showing its poor predictive and prognostic power 3, 4. In 2017 WHO published a new classification specific for pancreatic NENs, based on proliferation parameters (Ki‐67 and/or MI), and morphological features (well‐differentiated [WED] versus poorly differentiated [POD] tumors), and also on percentage of neuroendocrine component (at least 30% of the neoplasm) relative to the non‐neuroendocrine component 5. Thanks to this classification, WED‐NENs can be further divided into NET G1 (Ki‐67 < 3%; MI <2/10 high‐power fields [HPF]), NET G2 (Ki‐67 3–20%; MI 2–20/10 HPF) and NET G3 (Ki‐67 > 20%; MI >20/10 HPF); on the other hand POD tumors are classified as neuroendocrine carcinomas (NECs) G3, having by definition Ki‐67 > 20%. Further studies 3, 4, included in the European Neuroendocrine Tumor Society (ENETS) 2017 Guidelines on POD‐GEP neoplasms 6, revealed that a 55% Ki‐67 cut off can discriminate POD‐NENs with different median overall survival (OS) and response to therapy. Indeed, higher median OS in patients with Ki‐67 ≤ 55% is associated with lower responsiveness to platinum‐based therapy, which is conversely highly efficacious in POD‐NENs with Ki‐67 > 55% 7.

Current GEP‐NENs medical therapy includes somatostatin analogues (SSAs), targeted therapies (sunitinib and everolimus), peptide receptor radionuclide therapy and platinum‐based chemotherapy. These approaches aim directly at targeting tumor cells, thus promoting disease chronicity rather than regression 7. On the other hand, immunotherapy targeting immune checkpoints – which has shown efficacy in several other cancer types – only targets nonneoplastic elements of the tumor microenvironment, also known as immune context 8, thus inducing immune‐mediated regression of the tumor mass. Several studies, conducted in other cancer types, have suggested that patients responding to immunotherapy show abundant PD‐1^+^ T‐cell infiltration that co‐localizes with PD‐L1^+^ tumor or stromal cells 9. In contrast, poor response to immunotherapy is associated with modest expression of PD‐1/PD‐L1 and high nuclear levels of β‐catenin 9. Importantly, immunotherapy shows efficacy in other GEP neoplasms, which share the same mutational burden with GEP‐NENs 8, 9, 10, 11. Conversely, microenvironment and tumor inflammatory features (MoTIFs) in GEP‐NENs have been poorly investigated to date 12, 13, 14. We carried out extensive GEP‐NEN MoTIFs profiling, testing their relationship with the WHO classes and their potential prognostic relevance. Moreover, we built two MoTIFs‐based prognostic indices (PIs) for OS and disease‐free survival (DFS), and two nomograms including selected MoTIFs and clinical parameters.

## Materials and methods

### Study setting and design

This study was performed according to the clinical standards of the 1975 and 1983 Declaration of Helsinki and was approved by the Ethical Committee of Fondazione IRCCS Istituto Nazionale dei Tumori (INT) (No. INT 21/16). A prospectively‐maintained institutional clinical database from two Northern Italy (Milan) referral Centres for NET treatment (INT, and Humanitas Research Hospital – HRH) was retrospectively analyzed. The INT data were used to study the MoTIFs, and to develop the MoTIFs PIs and nomograms. The HRH series was used as an external validation set to assess the ability of the aforementioned prognostic tools to discriminate patients' prognosis.

### Patients

Information on consecutive adult (≥18 years) patients with NENs of any grade treated at INT and HRH from 1995 to 2015 with available tumor specimens (maximum 20 sections of 5 μm for each paraffin block) was extracted from the database. Of note, in 1995 SSAs and platinum‐based treatment were established as standard therapy for NENs: therefore, the choice has been made, taking into consideration that all the enrolled patients underwent surgery, followed by SSAs treatment for NETs G1–2, or chemotherapy for NETs G3 and NECs G3. The neuroendocrine nature was histologically confirmed in all specimens by immunohistochemistry (IHC) for chromogranin‐A and synaptophysin.

### IHC and MoTIFs scoring system

Biomarker expression was assessed by IHC in formalin‐fixed paraffin‐embedded tumor tissue sections following the manufacturer's instructions (see supplementary material, Table S1). The specificity of all reactions was checked, replacing the primary antibody with a nonrelated mouse immunoglobulin at comparable dilutions or using normal serum alone. Positive and negative controls were used as appropriate for each antibody, following the manufacturer's instructions. Sections were stained with antibodies to immune (CD3, CD4, CD8, PD‐1, PD‐L1, HLA‐I, HLA‐DR) and nonimmune markers (COX‐2, pS6, β‐catenin, NGFR, α‐SMA, CD31). All these proteins were evaluated in both neoplastic cells and stromal cells. To minimize assessment variability, IHC results for each protein (with the exception of β‐catenin, α‐SMA, CD31) were rendered semi‐quantitatively by adopting a scoring system taking into account both staining marker extent (% positive cells) and intensity. The expression (E) was defined as follows: up to 25% neoplastic cells, 1+; 26–50%, 2+; 51–75%, 3+; 76–100%, 4+. The immunostaining intensity (I) was ranked as low (1+; fainter than internal controls), normal (2+; as faint as controls), or strong (3+; more intense than controls). E and I were combined into a single score (S), calculated as E × I 15.

### Statistical analysis

In the whole cohort of INT and HRH patients, the expression of each of the MoTIFs in G1, G2 and G3 NEN subsets was compared by Kruskal–Wallis test followed by Dunn's multiple comparison test. The binary association between all the investigated features was investigated by Spearman's correlation coefficient.

The study endpoints were OS and DFS and univariable analyses were performed by estimating Kaplan–Meier curves, with the log‐rank test used to compare subgroups, and by fitting Cox models. The MoTIFs were modeled using a three‐knot restricted cubic spline 16; nonsignificant nonlinear terms were omitted.

Details of the methods used to develop PIs and nomograms are given in supplementary material, Supplementary materials and methods. In brief, based on INT patients' data we applied a methodology for selecting and combining the MoTIFs to construct PIs for OS and DFS 17; each selected MoTIF was categorized into two prognostic categories, and that associated with the worst prognosis was given a score of 1; the PI was the sum of the MoTIF scores. A backward selection procedure was applied to select the variables for inclusion in the multivariable Cox models used to develop the nomograms. The initial set included the end‐point‐specific MoTIF PIs and clinicopathological variables chosen *a priori*: patients' age, primary tumor site, morphology (WED; POD), Ki‐67 and β‐catenin (B0: absent; B1: cytoplasmic and/or membrane localization; B2: nuclear localization).

## Results

### Patients

Overall, 350 consecutively treated patients were included in the study, 314 from INT (G1: *n* = 89 [28.3%]; G2: *n* = 97 [30.9%]; G3: *n* = 128 [40.8%]) and 36 (all G3) from HRH (Table 1). Median follow‐up in the INT series was 84 months (interquartile range [IQR], 42–133); 141 patients died and 283 had disease recurrence as first event. All HRH patients died for the disease, and median time to death was 11 (7–69) months.

**Table 1:** Demographic, clinical, and pathological characteristics of the analyzed series

	INT	HRH
	*n* = 314	*n* = 36
Age (years)		
Median (first and third quartile)	59 (49–67)	61 (56–67)
Primary tumor site		
Foregut	85 (27.1)	17 (47.2)
Midgut	178 (56.7)	12 (33.3)
Hindgut	51 (16.2)	7 (19.4)
Morphology		
WED	210 (66.9)	3 (8.3)
POD	104 (33.1)	33 (91.7)
WHO grade		
G1	89 (28.3)	–
G2	97 (30.9)	–
G3	128* (40.8)	36 (100)

* G3 includes 104 NEC and 24 NET.

### IHC profiler of GEP‐NENs microenvironment: evolution in the immune‐ and inflammation‐related profile of tumor and stroma along with the NET to NEC transition

**Figure 1:**
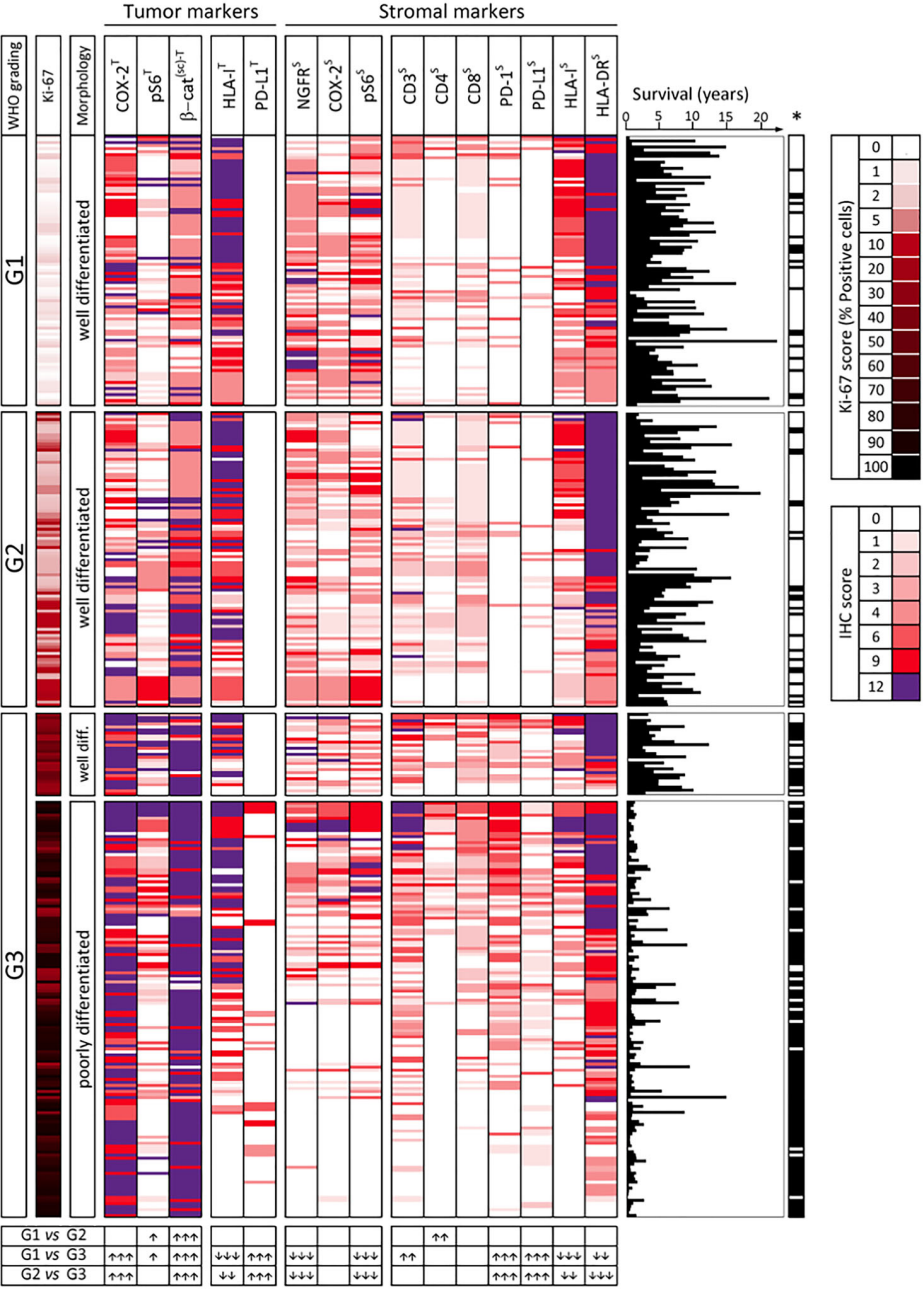
MoTIFs profile of 350 GEP‐NENs classified according to WHO grading, Ki‐67 score and morphology. Results of semi‐quantitative analysis (IHC scores) for expression of the indicated markers in each lesion is represented by the color code shown on the right‐hand side of the figure. Expression of each marker was evaluated in the tumor (superscript T) or in the stroma (superscript S). The β‐catenin^T^ IHC score reflects surface or cytoplasmic (s/c) staining. To aid the interpretation of data, within each subset defined by WHO grading, the tumor samples were ranked according to the sum of IHC score values of the immune‐related markers (HLA‐I^T^, PD‐L1^T^, CD3^S^, CD4^S^, CD8^S^, PD‐1^S^, PD‐L1^S^, HLA‐I^S^, HLA‐DR^S^). Therefore, lesions with the highest sum of these IHC scores are at the top of each grading subset. Ki‐67 score for each lesion was color coded as indicated in the legend on the right‐hand side of the figure. For each lesion a graph is shown indicating length of patient survival (years) and related death/censoring information (*Black: DOD; white: censored). Table at the bottom of the figure: expression of each marker was compared in the three main WHO grading subsets by Kruskal–Wallis test followed by Dunn's multiple comparison test. Up arrows and down arrows indicate increase or decrease of expression, respectively, in the subset with higher grading compared to the subset with lower grading. Number of arrows (1, 2, or 3) for each comparison reflects increasing significance (*p* < 0.05, *p* < 0.01, or *p* < 0.001, respectively).

Expression of immune, inflammatory and nonimmune markers was evaluated in tumor (coded by superscript ‘T’) and stromal (coded by superscript ‘S’) areas of the lesions. In the whole cohort of 350 patients (Figure 1 and see supplementary material, Figure S1). The overall immune and inflammation‐related profile of the GEP‐NENs showed highly significant changes in the transition from G1/G2 to G3 tumors, as documented by comparison of NETs and NECs for expression of each of the investigated markers, with the exception of COX‐2^s^ and of CD8^s^ (see table at the bottom of Figure 1). In detail, PD‐L1^T^ expression was found only in a subset of G3 NEC and PD‐L1^S^ increased in NECs compared to NETs. Overall PD‐L1 expression (% of cases with either PD‐L1^S^ or PD‐L1^T^ positivity at any level > 0) increased along the GEP‐NEN grading stages: from 8.99% in G1 and 12.37% in G2 to 37.04% in G3 WED and 48.91% in G3 POD. G3 NECs showed frequent loss of HLA‐I^T^, increased expression of CD31^T,S^ and of α‐SMA^T,S^ and (in 89/164 tumors) a transition to ‘nuclear staining only’ in the β‐catenin staining pattern (Figure 1 and see supplementary material, Figure S1).

Testing all the binary associations among the investigated markers, across the three grading subsets, provided further insight into the widespread phenotypic divergence between G1–2 NETs and G3 NETs and G3 NECs (see supplementary material, Figure S2). Collectively these results indicated that the transition from G1/G2 NETs to G3 NETs and G3 NECs is associated with profound changes in the tumor and stromal profile for inflammatory and immune‐related markers and point to more frequent activation of adaptive immunity in NECs (documented by increased CD3^s^, PD‐1^s^, and PD‐L1^s^) counteracted by strong immune escape mechanisms (HLA‐I^T^ loss), by expression of PD‐L1 on tumor or stroma, and by activation of inflammatory pathways involved in negative regulation of anti‐tumor immunity (enhanced COX‐2^T^ and both β‐catenin^s/c‐T^ and β‐catenin^n‐T^ expression) (see supplementary material, Figures S3 and S4).

### Selected immune‐related MoTIFs allow to build OS and DFS PIs

**Figure 2:**
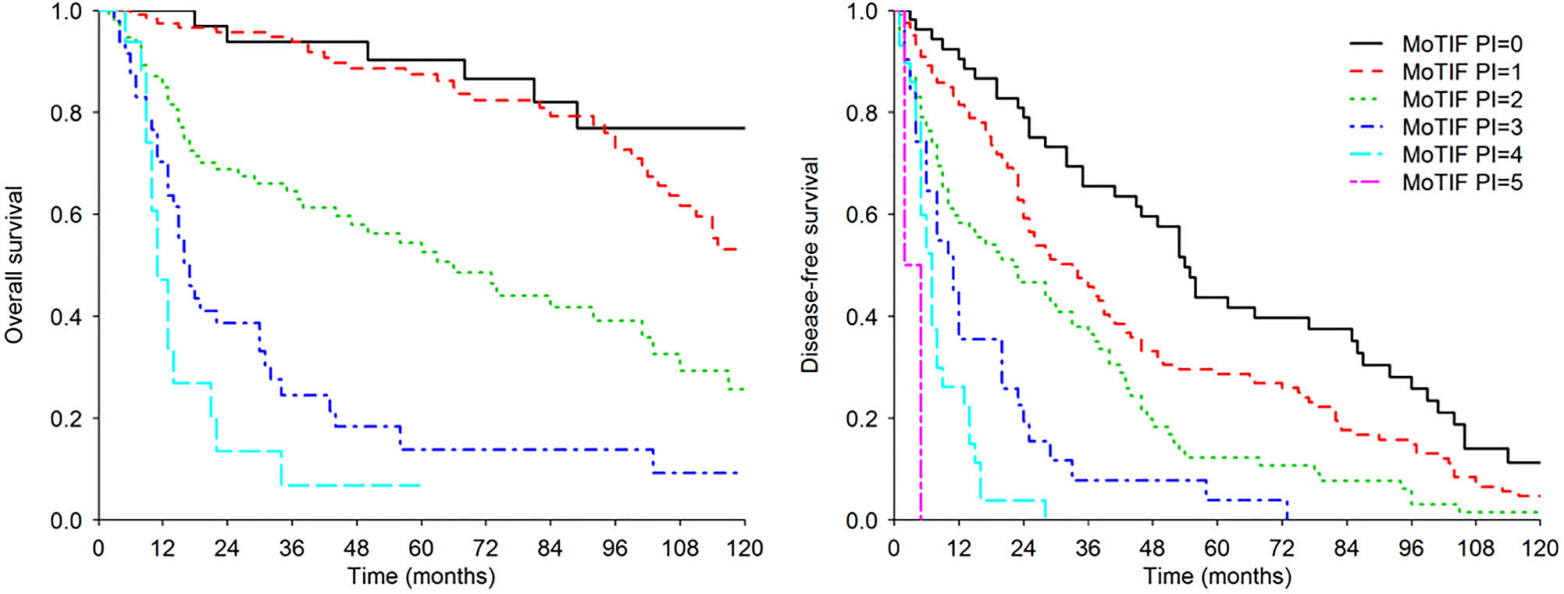
Kaplan–Meier curves for OS (left) and DFS (right) according to the PIs based on MoTIFs in the INT series. The MoTIFs PI for OS assumed values from 0 to 4, and the MoTIFs PI for DFS assumed values from 0 to 5.

The PIs were derived on the set of 314 INT patients. Univariable Cox analysis (see supplementary material, Table S2) showed significance of all MoTIFs but pS6^T^. High values of CD3^S^, CD4^S^, PD‐1^S^, PD‐L1^S^, COX‐2^T^, and pS6^T^ were associated with worse prognosis (hazard ratio [HR] estimates >1); conversely, high values of HLA‐I^T^, CD8^S^, HLA‐I^S^, HLA‐DR^S^, NGFR^S^, COX‐2^S^, and pS6^S^ were associated with good prognosis (HRs <1). The OS PI included four selected features, the positivity of which, associated with worse survival, was defined as follows: COX‐2^T^ > 4, PD‐1^S^ > 0, CD8^S^ < 1, and HLA‐I^S^ < 1. Figure 2 (left) shows the OS curves according to the PI. Using univariable Cox analysis the HR corresponding to a unit increment of the PI was 2.50 [95% confidence interval (CI), 2.12–2.96; Wald test *p* < 0.0001; the Harrell C statistic (C) (95% CI) = 0.761 (0.726–0.769) (optimism‐adjusted C = 0.761).

Even for DFS the PI methodology selected COX‐2^T^ and PD‐1^S^, together with other three immune features, HLA‐I^S^, HLA‐I^T^, and HLA‐DR^S^. Positivity was defined as follows: COX‐2^T^ > 4, PD‐1^S^ > 4, HLA‐I^S^ < 1, HLA‐I^T^ < 2, HLA‐DR^S^ < 6. Figure 2 (right) shows the Kaplan–Meier DFS curves according to the PI. The Cox model HR corresponding to a unit increment of the PI was 1.77 (95% CI, 1.58–1.99; Wald test *p* < 0.0001; C = 0.668 [0.637–0.699] [optimism‐adjusted C = 0.668]).

PIs composition according to the selected variables is represented in terms of scores in supplementary material, Tables S3 and S4, and in supplementary material, Figure S5 in terms of original semi‐quantitative values. In the HRH series we were able to verify the prognostic segregation operated by the MoTIFs PIs (see supplementary material, Supplementary materials and methods and supplementary material, Figure S6). These results suggest that increasing inflammation (tumor COX‐2), loss/downmodulation of HLA Class I molecules (even when expressed on stromal cells) and enhanced T cell functional impairment (PD‐1) have a significant and negative impact on both OS and DFS.

### Morphology and Ki‐67 have their main prognostic impact on OS, while DFS is associated with morphology and selected immune‐related MoTIFs

**Figure 3:**
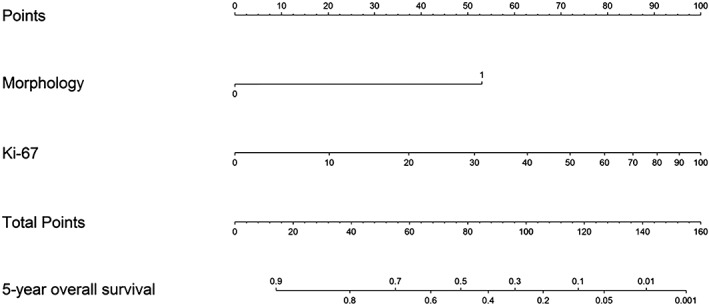
Nomogram to predict 5‐year OS. The nomogram was derived from a multivariable Cox model including the two selected variables, morphology and Ki‐67. Instructions: the nomogram provides a method of calculating 5‐year OS probability on the basis of a patient's combination of covariates. Locate the tumor Ki‐67 value, draw a line straight upwards to the Points axis to determine the score associated with Ki‐67. Do the same for morphology, sum the two scores and locate the total score on the total points axis. Draw a line straight downwards to the 5‐year OS axis to obtain the probability.

OS and DFS curves according to grade or morphology are shown in supplementary material, Figure S7. Morphology segregated two groups with very divergent OS and DFS, with worse prognosis associated with POD tumors. However, G1‐2 (Ki‐67 ≤ 20%) patients shared similar OS. For this reason, in the following analyses, we evaluated Ki‐67 as a continuous variable in order to exploit its informative content. By univariable Cox analysis (see supplementary material, Table S4), morphology and Ki‐67 showed stronger association (higher HR estimates) than the MoTIF variables with both OS and DFS; they were selected by the backward procedure and were included in the Cox model used to generate the OS nomogram (Table 2). We explored whether the prognostic effect of one variable could vary at different levels of the other, but no significant results were obtained (*p* for interaction = 0.222). The nomogram allows 5‐year OS prediction according to specific morphology and Ki‐67 values, and it is useful for exploiting the information given by Ki‐67 as a continuous variable (Figure 3, and supplementary material, Figure S8 for nomogram‐predicted OS in WED and POD subsets). Based on the above results, we also derived an OS stratification of G3 patients into three groups (see supplementary material, Figure S9): better prognosis, Ki‐67 ≤ 55%, WED; intermediate prognosis, Ki‐67 ≤ 55%, POD; worse prognosis, Ki‐67 > 55%, POD.

**Table 2:** Results of the multivariable Cox models for OS and DFS used to derive the nomograms

	HR	95% CI	*P* value
OS model			
Ki‐67*			<0.001
70.0% versus 1.8%	11.32	(5.28–24.24)	
Morphology			<0.001
POD versus WED	4.83	(2.30–10.15)	
DFS model			
Morphology			<0.001†
POD versus WED with MoTIFs PI = 0	10.23	(5.67–18.47)	
POD versus WED with MoTIFs PI = 5	2.87	(1.21–6.81)	
MoTIF PI			<0.001†
5 versus 0 with morphology WED	6.21	(2.52–13.31)	
5 versus 0 with morphology POD	1.74	(0.70–4.30)	

* Fitted through 3‐knots restricted cubic spline; the two values are, respectively, the third and first quartile of Ki‐67 distribution.

† Wald test *P* value of the main effect and interaction between morphology and MoTIF PI.

**Figure 4:**
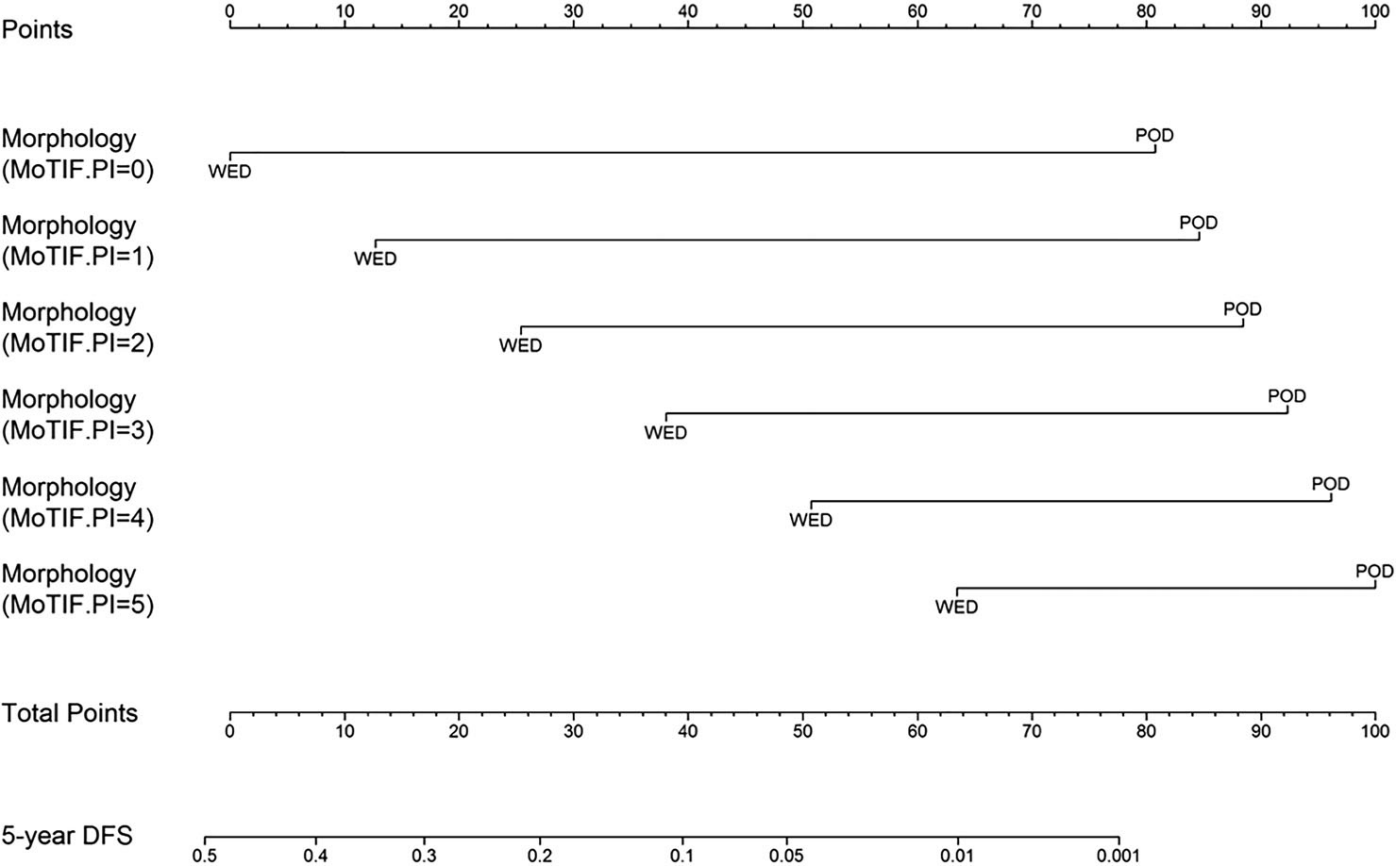
Nomogram to predict 5‐year DFS. The nomogram was derived from a multivariable Cox model including the two selected variables, morphology and MoTIFs PI, together with their interaction. Instructions: the nomogram provides a method of calculating 5‐year DFS probability on the basis of a patient's combination of covariates. Locate the axis corresponding to morphology and MoTIFs PI value and draw a line straight upwards to the points axis corresponding to that combination. Draw a line straight downwards to the 5‐year DFS axis to obtain the probability.

In the multivariable DFS Cox model, the backward procedure selected morphology and MoTIFs PI (including COX‐2^T^, PD‐1^S^, HLA‐I^S^, HLA‐I^T^, and HLA‐DR^S^). The interaction between the two variables was not statistically significant, but achieved a *P* value as low as 0.050, disclosing a different prognostic effect of morphology at different levels of PI or *vice versa*: at increasing PI the importance of morphology decreased (Table 2, HR = 10.23 for PI = 0 versus HR = 2.87 for PI = 5). Conversely, the PI was more able to segregate WED (HR = 6.21) than POD patients' prognosis (HR = 1.74). Thus, we decided to retain such interaction in the final Cox model used to generate the DFS nomogram (Table 2 and Figure 4). The interaction effect is clearer in the nomogram (Figure 4); the morphology effect is represented by the length of the axis (the longer the greater), and is greater at low PI levels (the longest axis corresponds to PI = 0) and decreases at increasing PI (the shortest axis corresponds to PI = 5). The nomogram calibration plots are shown in supplementary material, Figure S10; the nomogram discriminative ability was very good for OS (*C* = 0.860 [0.838–0.882]; optimism‐adjusted *C* = 0.860), and was slightly lower for DFS (*C* = 0.732 [0.705–0.758]; optimism‐adjusted *C* = 0.731). In the HRH series we were able to verify the prognostic segregation operated by the nomograms (see supplementary material, Supplementary materials and methods and supplementary material, Figure S11).

Taken together these results suggest that DFS may be improved by preexisting immunity (which explains the impact of immune‐related MoTIFs on the DFS model). On the other hand, OS appears to be mainly associated with tumor‐intrinsic biological aggressiveness (marked by POD morphology and high Ki‐67), features that can no longer be counteracted even by a preexisting spontaneous immune response.

## Discussion

The quest for developing an improved classification of GEP‐NEN, and specifically of the POD NEC G3 subset, stems from two distinct but overlapping needs, related to prognosis and treatment. On one hand, as outlined in the recent ENETS consensus guidelines 6, the available evidence suggests that POD NENs (NECs G3) are a heterogenous set of tumors, whose complexity and prognosis are not fully dissected by the available classification parameters (Ki‐67 and morphology). On the other hand, advanced NENs remain poorly responsive to conventional (chemotherapy) or targeted approaches. Thus, there is an urgent need to identify new biological or molecular markers defining previously undetected subsets of advanced NEN patients who may be potentially responsive to innovative treatments such as immunotherapy 18.

In this study we found that the immune‐related profile of GEP‐NENs shows a clear shift at the G1/G2–G3 transition (both NETs and NECs). This grading‐related evolution of the GEP‐NEN immune profile suggested promotion of adaptive immunity in a subset of G3 NEC being counteracted by immune escape mechanisms (HLA‐I^T^ loss) and by promotion of inflammatory mechanisms that negatively regulate adaptive immunity (COX‐2^T^ and β‐catenin^T^). Tumor and microenvironment immune profiling in the G3 subset allowed the identification of two groups: patients with reduced expression of HLA‐I^T^, associated with reduction of lymphoid markers, CD3^S^ and CD8^S^ and loss of PD‐L1^S^ (these patients have the worst prognosis and appear less suitable for immunotherapy (see supplementary material, Figure S3). On the other hand, patients with retention of expression of HLA‐I^T^ and the presence of a lymphoid infiltrate (CD3^S^, CD8^S^, PD‐L1^S^) have a more favorable prognosis and could potentially be responsive to immunotherapy (see supplementary material, Figure S3) 18, 19.

A weak but significant inverse correlation was found between Ki‐67 and HLA‐I^T^ (*r* = −0.180) and between Ki‐67 and CD8^S^ (*r* = −0.293), while PD‐L1^T^ and Ki‐67 showed a direct correlation (*r* = 0.280). These findings suggest that increased tumor grading (captured by Ki‐67) is associated also with impairment of anti‐tumor immunity through HLA‐I downmodulation, reduced CD8 infiltration and enhanced PD‐L1 expression on tumor cells. We also studied MoTIFs prognostic value and generated OS and DFS PIs based on selected MoTIFs able to stratify patients' prognosis. The negative impact on DFS of COX‐2^T^ and PD‐1^S^ and the positive impact of HLA‐I^S^, HLA‐I^T^, and HLA‐DR^S^ have a potentially straightforward interpretation: COX‐2^T^ has been shown to mediate inhibition of type‐I interferon (IFN) and T cell‐mediated anti‐tumor responses 20, while a high expression of PD‐1 can mark functionally impaired (exhausted) T cells at tumor sites 21. In contrast, retained HLA‐ Class I and Class‐II expression in the tumor microenvironment are essential requisites for tumor‐antigen recognition by CD8^+^ and CD4^+^ T cells 22, 23. Therefore, the specific MoTIFs selected by the DFS model strongly point to the relevance of a functional adaptive immune response in delaying tumor relapse.

Finally, two nomograms were elaborated based on MoTIFs and WHO prognostic parameters for estimating 5‐year OS and DFS probability. The nomogram findings showed that the combination of morphology and Ki‐67 is the best prognosticator of OS in NENs. Moreover, the association of POD morphology and a Ki‐67 threshold of 55% enables the identification of three G3 subpopulations with different OS, in line with previous studies 3, 4. The analysis of tumor microenvironment showed that no biomarker was relevant enough to modify the prognostic value of the WHO 2017 classification 5. Anyway, these results suggest that DFS may be improved by preexisting immunity, which explains the impact of immune‐related MoTIFs on the DFS model, while OS appears to be mainly associated with tumor‐intrinsic biological aggressiveness (marked by POD morphology and high Ki‐67), features that can no longer be counteracted even by a preexisting spontaneous immune response.

This study has some limitations. First, the HRH series only included G3 (both NETs and NECs) patients, thus the external validity of our tools should be demonstrated on G1‐2 patients. Second, although we showed different levels of MoTIFs association according to grade, in the absence of a large G3 population we could not generate G3‐specific prognostic tools.

In conclusion, this study shows that microenvironment‐related immune and inflammatory markers can improve prognostic prediction in GEP‐NENs, when combined with established Ki‐67 and morphology parameters. Moreover, at least a subset of G3 POD (NECs) has microenvironment features consistent with spontaneous activation of adaptive immunity (co‐expression of CD3, CD4, CD8, PD‐1, and PD‐L1), suggesting potential for responsiveness to immunotherapy targeting immune checkpoints.

## Author contributions statement

MM and AA conceived and designed the study. MM, RM, and AA developed the methodology. AP, PS, GT, GC, CP, SP, CC, VM, and GS acquired data (provided animals, acquired and managed patients, provided facilities, etc.). RM and FB analyzed and interpreted data (e.g. statistical analysis, biostatistics, computational analysis). MM, RM, AM, and AA wrote, reviewed and/or revised the manuscript. KK, GC, LG, and AC provided administrative, technical or material support (i.e. reporting or organizing data, constructing databases). MM, RM, AM, FdeB, GP, and AA supervised the study.

## Supporting information


**Supplementary materials and methods**

**Figure S1.** Nuclear β‐catenin, CD31, and α‐SMA profile of 350 GEP‐NENs classified according to WHO grading, Ki‐67 score and morphology
**Figure S2.** Correlation analysis of all MoTIFs markers in 350 GEP‐NENs classified according to WHO grading
**Figure S3.** Immunohistochemistry analysis of NEC G3 showing reduced expression of HLA‐I^T^
**Figure S4.** Immunohistochemistry analysis of NEC G3 showing retention of expression of HLA‐I^T^
**Figure S5.** Heatmaps showing the OS (left) and DFS (right) PIs composition according to the selected MoTIFs represented as semi‐quantitative values from 0 to 12
**Figure S6.** OS and DFS Kaplan–Meier curves estimated on the HRH series according to the PIs values
**Figure S7.** Kaplan–Meier curves for OS and DFS according to grade (G1, G2, G3) and morphology (WED, POD)
**Figure S8.** Nomogram predicted 5‐year OS according to Ki‐67 and morphology
**Figure S9.** Kaplan–Meier curves of OS in G3 patients according to Ki‐67 and morphology
**Figure S10.** Calibration plot for of the 5‐year OS and DFS nomograms on the INT series
**Figure S11.** Kaplan–Meier curves for OS and DFS according to the nomogram predictions on the HRH series

**Table S1.** Antibody sources and dilutions
**Table S2.** Univariable Cox model results for OS and DFS
**Table S3.** Composition of MoTIFs PI for OS according to the selected variables COX‐2^T^, PD‐1^S^, CD8^S^, and HLA‐I^S^
**Table S4.** Composition of MoTIFs PI for DFS according to the selected variables COX‐2^T^, PD‐1^S^, HLA‐I^S^, HLA‐I^T^, and HLA‐DR^S^
