# Supplementary material for: Microenvironment and tumor inflammatory features improve prognostic prediction in gastro‐entero‐pancreatic neuroendocrine neoplasms
Source: J Pathol Clin Res. 2019 Jul 9;5(4):217–26. doi: 10.1002/cjp2.135 (PMC6817832; doi:10.1002/cjp2.135)
Supplement: Supplementary file 1 — Supplementary materials and methods [file CJP2-5-217-s001.docx]

**Microenvironment and tumor inflammatory features improve prognostic prediction in gastro-entero-pancreatic neuroendocrine neoplasms**

Milione M *et al* *J Pathol Clin Res* DOI: 10.1002/cjp2.135

**Supplementary materials and methods (reference numbers refer to the main text)**

**Prognostic indexes (PIs) for overall survival (OS) and disease-free survival (DFS) combining the semi-quantitative microenvironment and tumor inflammatory features (MoTIFs)**

OS time was calculated from the date of diagnosis to the date of death from all causes, with censoring at the date of last follow-up in living patients. DFS time was calculated from the date of diagnosis to the date of first event (local or distant tumor relapse or death without relapse, whichever occurred first), with censoring at the date of last follow-up in patients alive and without event.

We applied a methodology for adaptively constructing PIs for OS and DFS combining semi-quantitative MoTIFs [17]. The procedure allowed to select the MoTIFs and categorize the selected ones by determining a set of cut-off based binary rules for each feature, whereby positivity is associated with worse prognosis (for instance, if COX-2^T^ assumes high values with worse prognosis, the binary rule will be “COX-2^T^>cut-off results positive and scores 1” and “COX-2^T^ equal or less than the cut-off results negative and scores 0”). Moreover, the features are ordered according to their prognostic value, and the features to be included in the PI are selected by deriving a cross-validated optimal number of features (*q*). However, the randomly partitioned dataset for cross-validation and, as a consequence, the *q* value, depend upon the random seed. Thus, we applied the procedure 1000 times using different random seeds and choose *q* as the mode of the distribution of the cross-validated *q_i_* (i= 1, ...1000). For each patient, the PI was obtained as the sum of the selected feature scores, thus its possible values varied from 0 to the number of selected features.

**Nomograms for OS and DFS**

For each end-point, the variables selected after applying the AIC-based backward procedure were included in the multivariable Cox models from which the nomograms to predict 5-year OS or DFS were developed [24].

Cox model performance was assessed in the Istituto Nazionale dei Tumori (INT) development cohort by calibration plot as indicator of internal calibration and Harrell C statistic and its 95% confidence interval as measure of discriminative ability [25]. A bootstrap procedure was also used to adjust the C estimate for the optimism implicit in the use of the same dataset for model-fitting and performance evaluation [26]. Ki-67 was modeled as continuous variable using a three-knot restricted cubic spline [16]; non-significant nonlinear terms were omitted.

The analyses were performed with SAS (Cary, NC, USA) and R software [R Core Team (2016): R: A language and environment for statistical computing. R Foundation for Statistical Computing, Vienna, Austria. URL [*https://www.R-project.org/*](https://www.R-project.org/)*.* Last accessed October 2018].

**Results on the Humanitas Research Hospital series**

The Humanitas Research Hospital (HRH) series was used to assess discriminative ability [(Harrell C statistics and its 95% confidence interval (CI)] of the prognostic instruments generated in the present work. By means of these data we were able to verify the prognostic segregation operated by the PIs and nomograms on a G3 series.

**MoTIFs PIs for OS and DFS**

In Figure S6 we show the OS and DFS Kaplan-Meier curves estimated on the HRH series according to the PIs values. The PIs were categorized in two groups (1-2 *vs* 3-4); no patients achieved a score of 5 for the DFS PI. Despite of the low Harrell C figures [OS 0.555 (0.473-0.637); DFS 0.581 (0.454-0.709)], even in the HRH series of G3 patients the PIs were able to operate survival curves segregation especially for OS, in that the curves corresponding to lower scores were well separated from the higher score’s curves.

**OS and DFS nomograms**

By categorizing the nomogram predicted 5-year OS of HRH series patients by quartiles (9 patients in each) and estimating the actual OS in the 4 groups by the Kaplan-Meier method, the predicted OS ordering was preserved by actual OS. Indeed, in the Supplementary figure 11 (left) the worst prognosis group was Q1 (predicted OS <0.0048; red line), followed by Q2 (predicted OS ≥0.0048 and <0.0087; green line), Q3 (predicted OS ≥0.0087 and <0.0873; blue line) and Q4 (≥0.0873; cyan line) [Harrell C=0.690 (0.591-0.788); 95%CI].

The DFS nomogram predicted probabilities were very low, all ≤0.2% but 3, equal to 4%, 11% and 34%). Thus, we used a three-categories classification according to the tertiles (Figure S11, right). In this case, the two groups with the lower predicted DFS (T1 and T2; red and green lines) had similar actual DFS, and the third one (T3; blue line) achieved the highest actual DFS. [Harrell C=0.661 (0.536-0.786); 95% CI].
