# Supplementary material for: Microenvironment and tumor inflammatory features improve prognostic prediction in gastro‐entero‐pancreatic neuroendocrine neoplasms
Source: J Pathol Clin Res. 2019 Jul 9;5(4):217–26. doi: 10.1002/cjp2.135 (PMC6817832; doi:10.1002/cjp2.135)
Supplement: Supplementary file 2 — Figure S1. Nuclear β‐catenin, CD31, and α‐SMA profile of 350 GEP‐NENs classified according to WHO grading, Ki‐67 score and morphology Figure S2. Correlation analysis of all MoTIFs markers in 350 GEP‐NENs classified according to WHO grading Figure S3. Immunohistochemistry analysis of NEC G3 showing reduced expression of HLA‐IT Figure S4. Immunohistochemistry analysis of NEC G3 showing retention of expression of HLA‐IT Figure S5. Heatmaps showing the OS (left) and DFS (right) PIs composition according to the selected MoTIFs represented as semi‐quantitative values from 0 to 12 Figure S6. OS and DFS Kaplan–Meier curves estimated on the HRH series according to the PIs values Figure S7. Kaplan–Meier curves for OS and DFS according to grade (G1, G2, G3) and morphology (WED, POD) Figure S8. Nomogram predicted 5‐year OS according to Ki‐67 and morphology Figure S9. Kaplan–Meier curves of OS in G3 patients according to Ki‐67 and morphology Figure S10. Calibration plot for of the 5‐year OS and DFS nomograms on the INT series Figure S11. Kaplan–Meier curves for OS and DFS according to the nomogram predictions on the HRH series [file CJP2-5-217-s002.docx]

**Microenvironment and tumor inflammatory features improve prognostic prediction in gastro-entero-pancreatic neuroendocrine neoplasms**

Milione M *et al. J Pathol Clin Res* DOI: 10.1002/cjp2.135


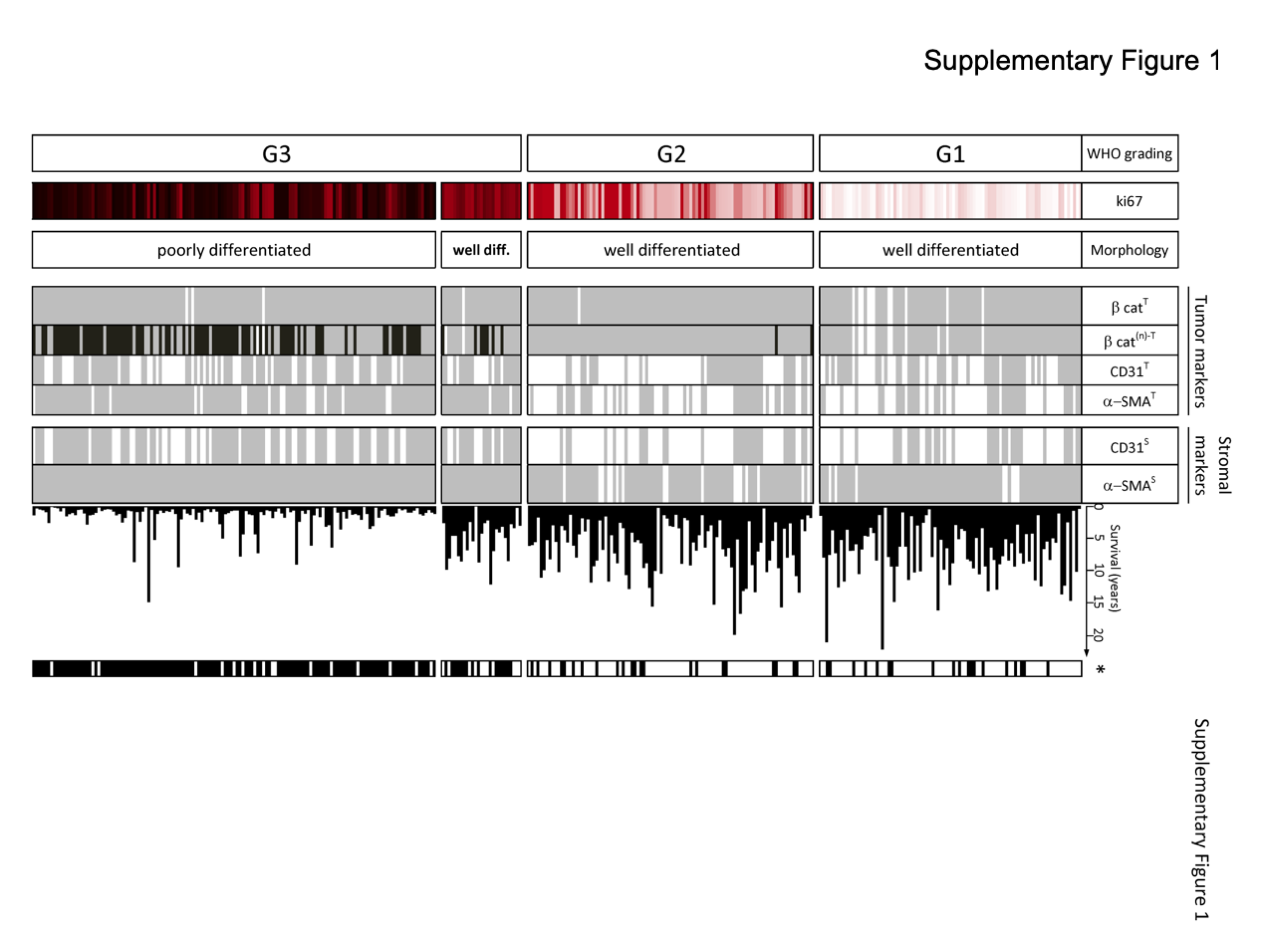


**Figure S1. Nuclear β-catenin, CD31 and α-SMA profile of 350 GEP-NENs classified according to WHO grading, Ki-67 score and morphology.**

The same set of lesions shown in Figure 1 was characterized for expression of the indicated markers. T and S superscripts indicate marker assessment in the tumor parenchyma or in the associated stroma. Expression of the indicated markers in each lesion is color coded as follows: white: marker not expressed; grey: marker expressed, black: nuclear staining. Color coding for Ki-67 score and graphs indicating length of patient’s survival as in Figure 1. Lesions in each grading subset are listed in the same sequential order as in Figure 1.

Abbreviations: GEP-NENs, gastro-entero-pancreatic neuroendocrine neoplasms; WHO, World Health Organization.


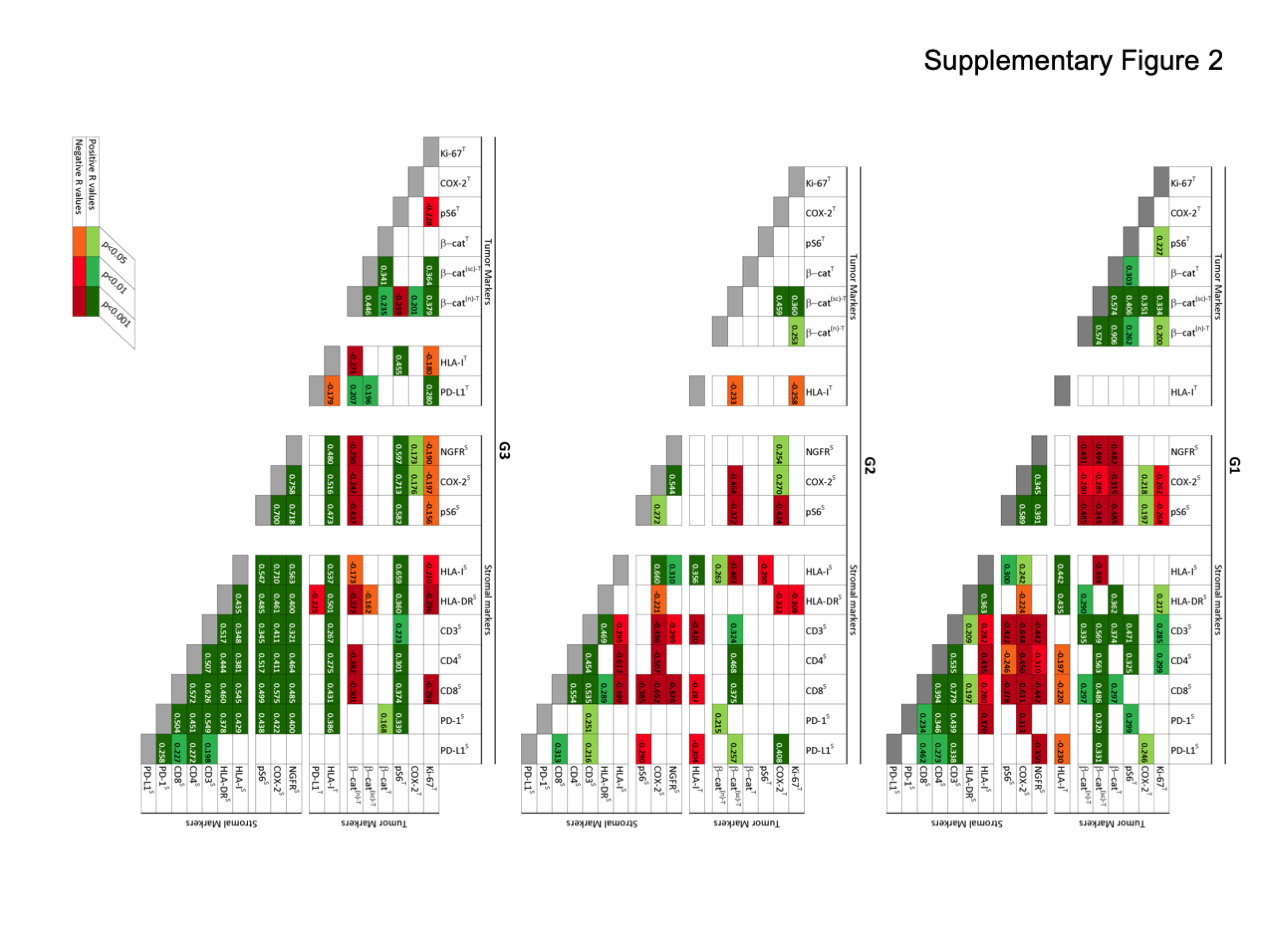


**Figure S2. Correlation analysis of all MoTIFs markers in 350 GEP-NENs classified according to WHO grading.**

Binary associations of all investigated markers were tested in the G1, G2 and G3 subsets by Spearman correlation analysis. Only significant *r* values (either positive or negative) are shown. Corresponding *p* values are represented by the color code indicated at the bottom of the Figure. S, sc, n and T superscripts of the indicated markers are as in the legends to Figure 1 and Figure S1. Immune-related markers (CD3^s^, CD4^s^, CD8^s^, PD-1^s^ and PD-L1^s^) showed significant and positive correlations with β-catenin^s/c-T^ and/or β-catenin^n-T^ in the G1 NETs, but were not correlated or showed a negative correlation with β-catenin^s/c-T^ and/or β-catenin^n-T^ in the G3 NETs and G3 NECs. HLA-I^T^ was positively correlated with most stromal immune markers (CD3^s^, CD4^s^, CD8^s^, PD-1^s^) in the G3 NECs, but not in G1 NETs. Opposite correlations were observed in the G1 NETs compared to G3 NETs and G3 NECs between stromal inflammatory (NGFR^s^, COX-2^s^ and pS6^s^) and immune CD3^s^, CD4^s^, CD8^s^ markers and PD-1^s^. On the other hand, G1–2 (NETs) and G3 (NETs and NECs) also shared several similar binary associations, including the positive correlations of β-catenin (either s/c-T or n-T) with Ki-67 and of PD-L1^s^ with CD3^s^, CD4^s^, CD8^s^ and PD-1^s^.

Abbreviations: MoTIFs, microenvironment and tumor inflammatory features; GEP-NENs, gastro-entero-pancreatic neuroendocrine neoplasms; WHO, World Health Organization.


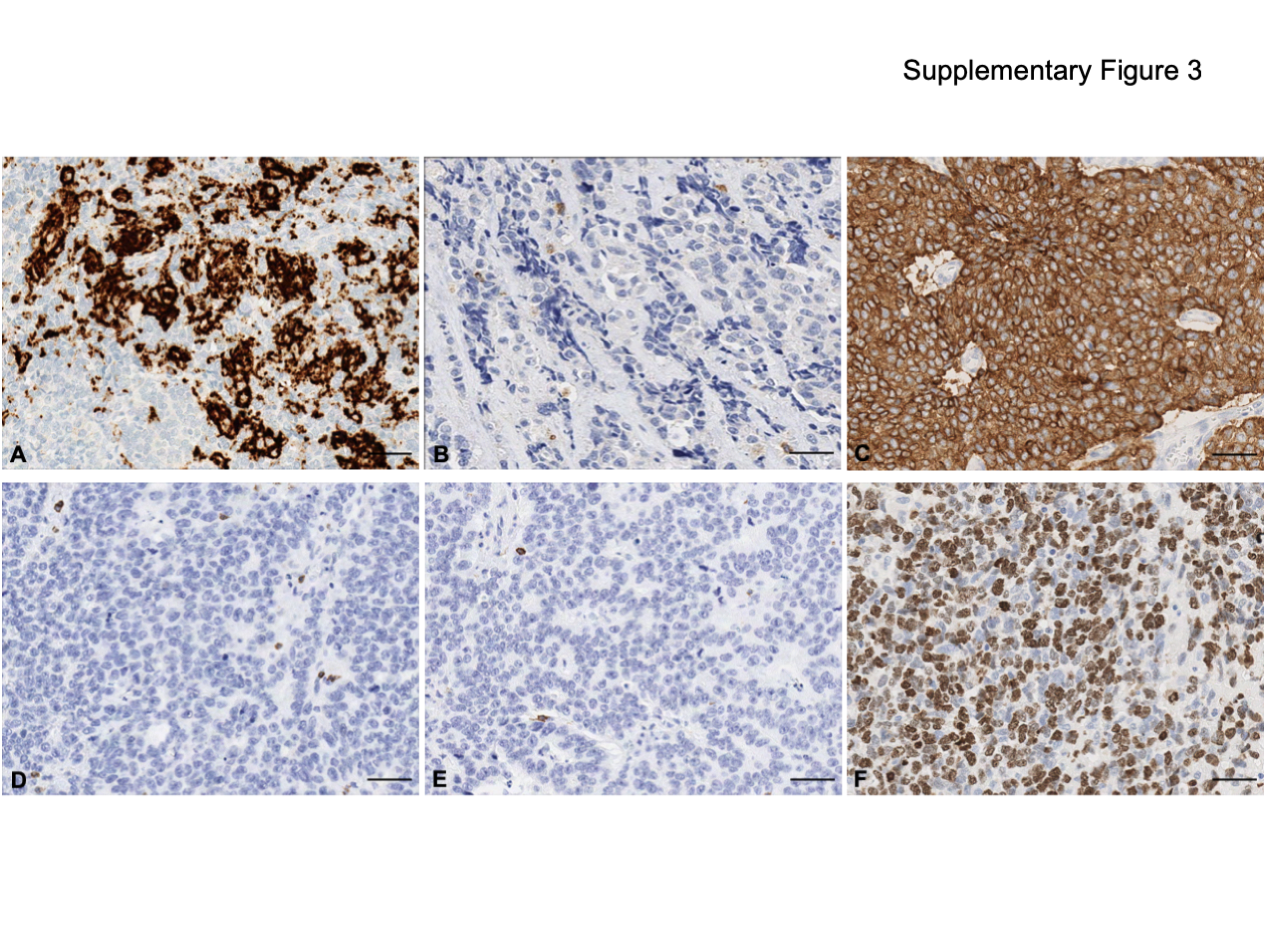
**Figure S3. Immunohistochemistry analysis of NEC G3 showing reduced expression of HLA-I^T^.**

A: PD-L1^T^; B: HLA-I^T^; C: Synaptophysin; D: CD3^T^; E: CD8^T^; F: Ki-67^T^; Scale bars = 50 µm. 20x objective magnification


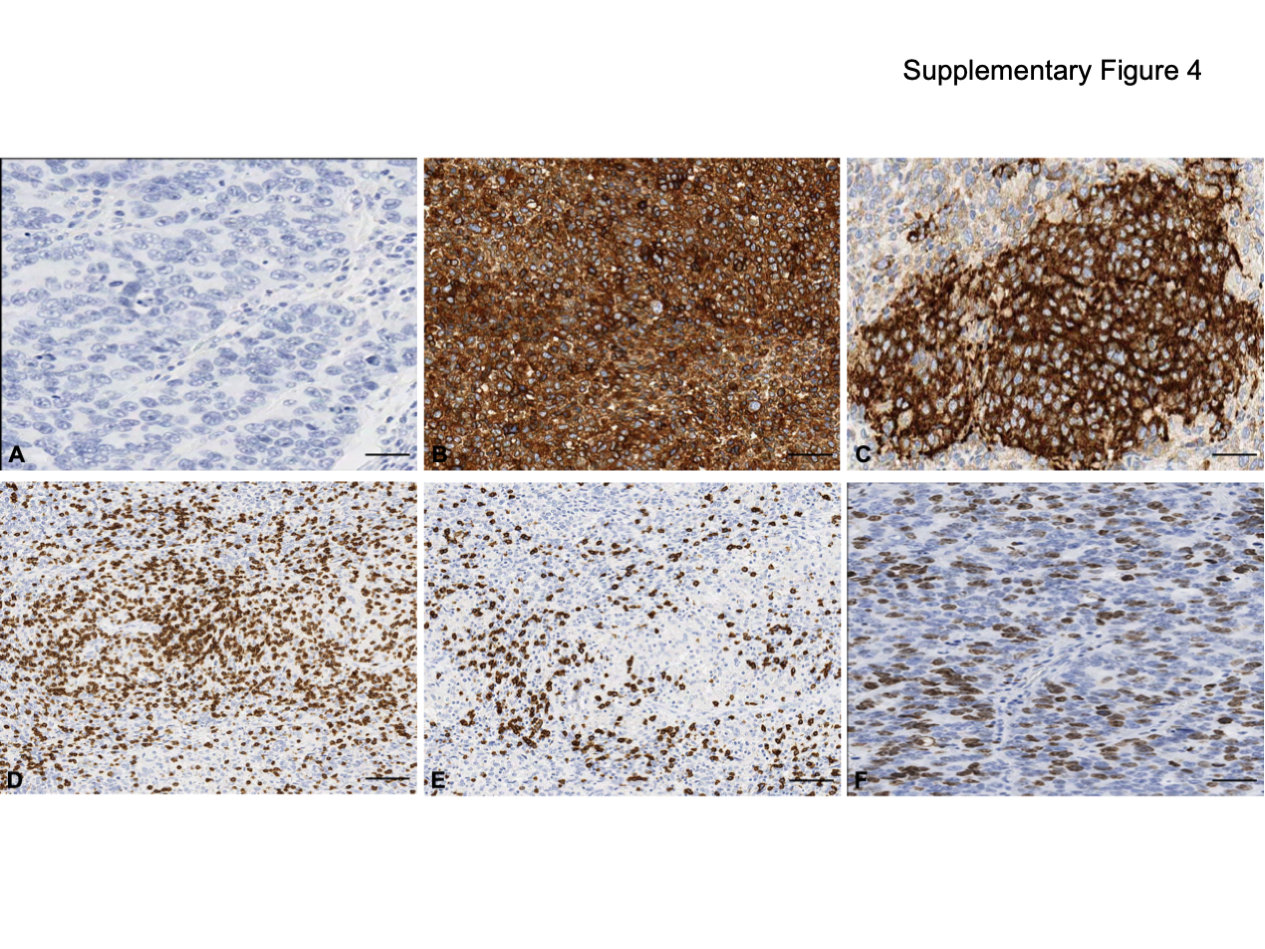


**Figure S4. Immunohistochemistry analysis of NEC G3 showing retention of expression of HLA-I^T^.**

A: PD-L1^T^; B: HLA-I^T^; C: Synaptophysin; D: CD3^T^; E: CD8^T^; F: Ki-67^T^; Scale bars = 50 µm. 20x objective magnification.


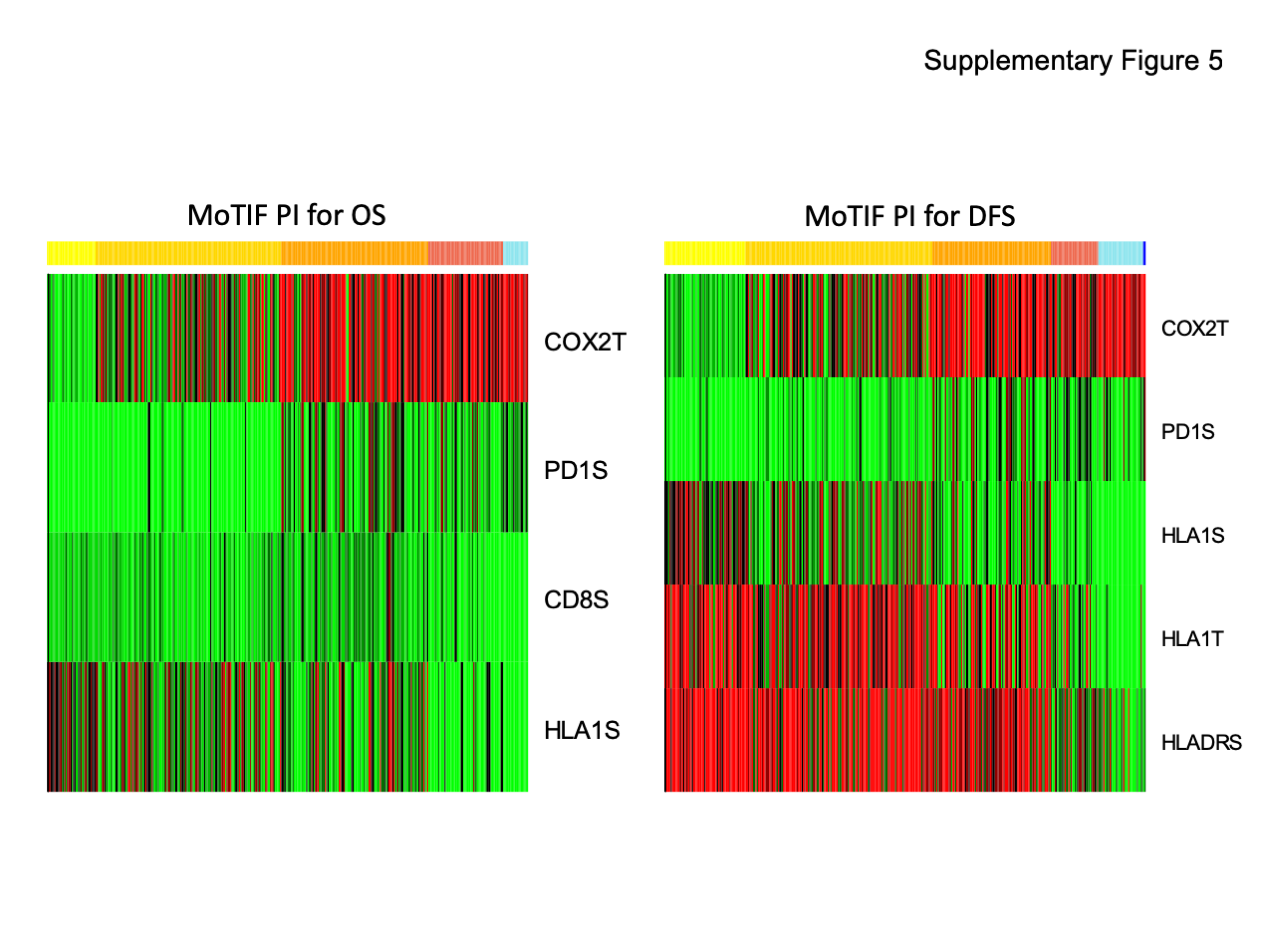


**Figure S5. Heatmaps showing the OS (left) and DFS (right) PIs composition according to the selected MoTIFs represented as semi-quantitative values from 0 to 12.**

Horizontal sections: MoTIFs included in the OS (left) or DFS (right) PIs. Vertical sections: patients ordered according to PIs values. *Color keys:* MoTIFs (grades of green, black and grades of red represent increasing values from 0 to 12); PIs (0 yellow, 1 light orange, 2 dark orange, 3 coral, 4 cyan, 5 blue). Unfavorable OS groups (PI= 3, 4) had a greater prevalence of high COX-2^T^ and PD-1^S^ values and low CD8^S^ and HLA-I^S^. Unfavorable DFS groups (PI= 3, 4, 5; 2 pts only in the last group) had a greater prevalence of high COX-2^T^ and PD-1^S^ values, and low HLA-I^S^, HLA-I^T^, and HLA-DR^S^ (see also Tables S2 and S3).

Abbreviations: OS, overall survival; DFS, disease-free survival; PIs, prognostic indexes; MoTIFs, microenvironment and tumor inflammatory features.


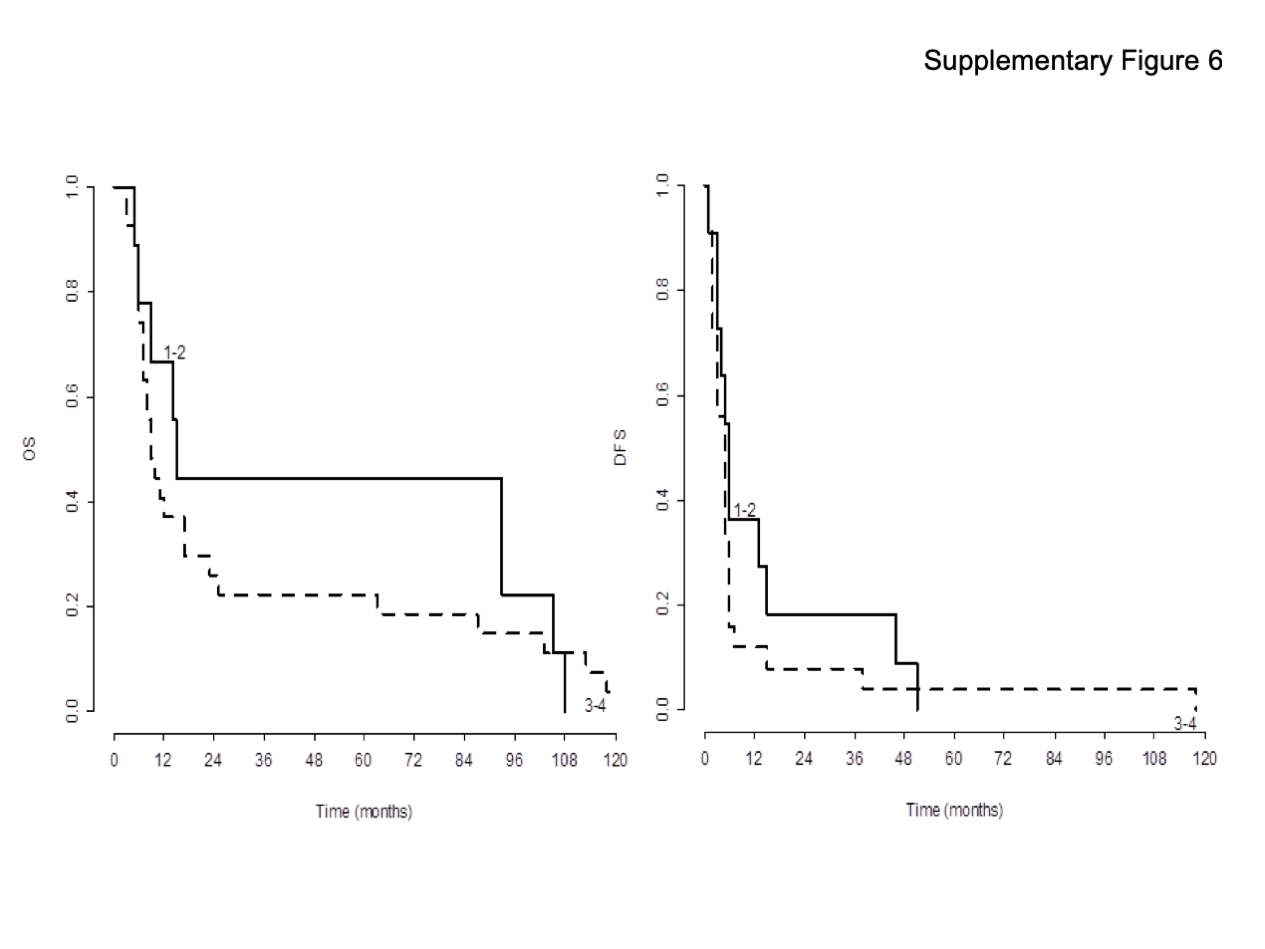


**Figure S6. OS (left) and DFS (right) Kaplan-Meier curves estimated on the Humanitas Research Hospital series according to the PIs values.**

Abbreviations: OS, overall survival; DFS, disease-free survival; HRH, Humanitas Research Hospital; PIs, prognostic indexes.


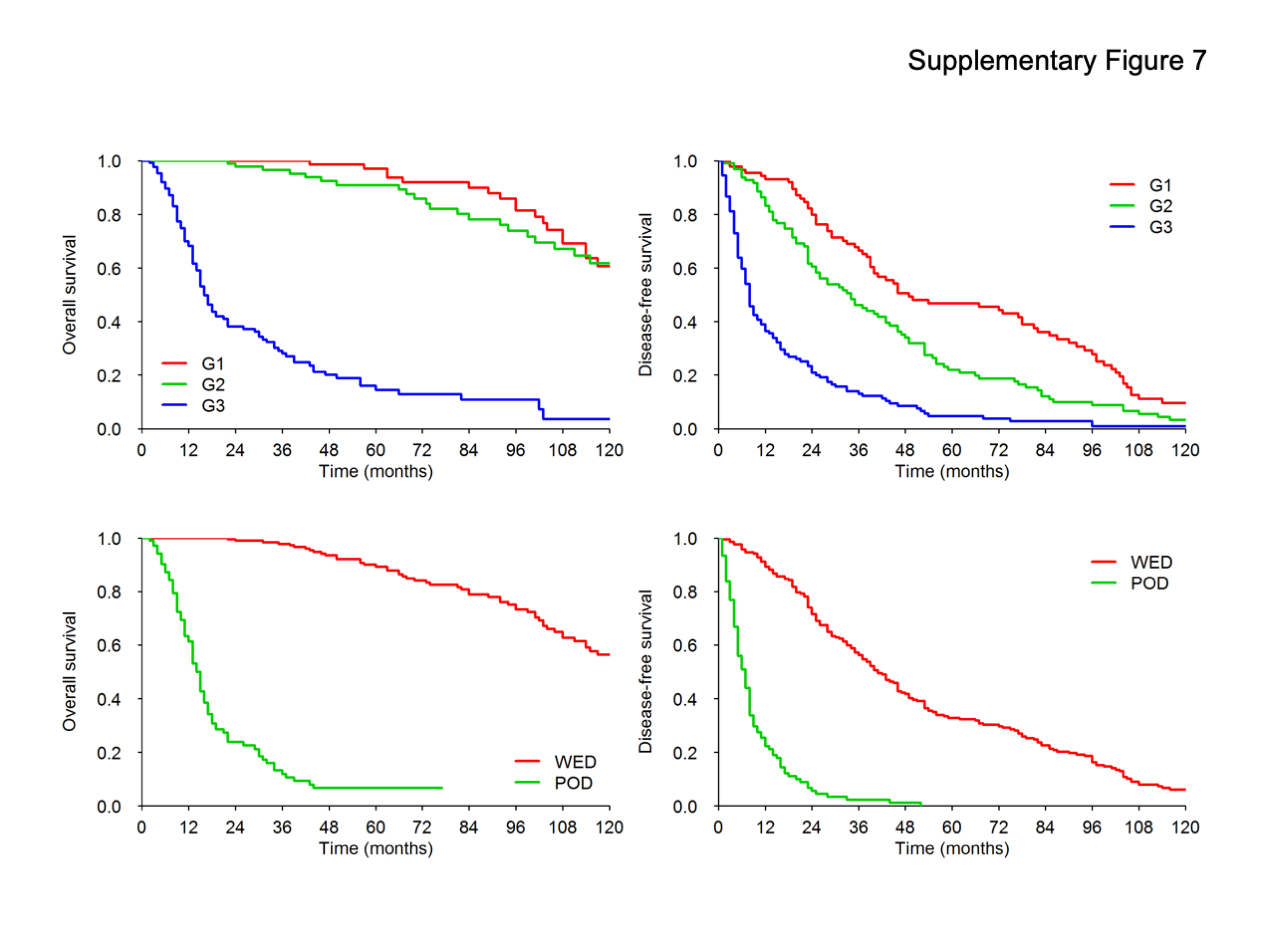


**Figure S7. Kaplan-Meier curves for OS (left) and DFS (right) according to grade (G1, G2, G3) and morphology (WED, POD).**

Abbreviations: OS, overall survival; DFS, disease-free survival; WED, well-differentiated; POD, poorly-differentiated.


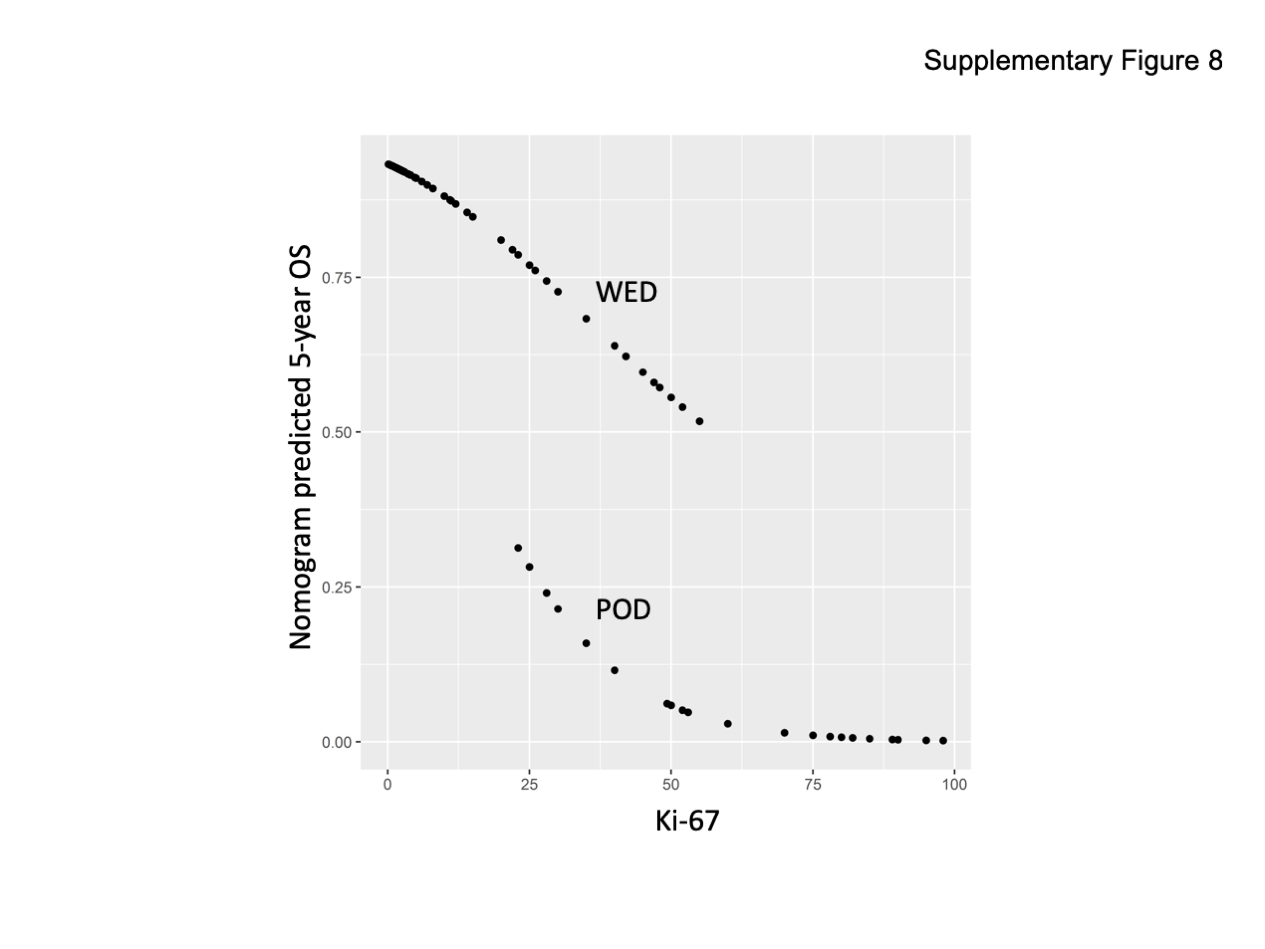


**Figure S8. Nomogram predicted 5-year OS according to Ki-67 and morphology.**

Morphology segregates two groups at better (WED; upper curve) and worse prognosis (POD; lower curve) and Ki-67 is able to refine OS within each of the two groups. For WEDs, the association between Ki-67 and OS was remarkable throughout the Ki-67 range, with rapid OS decrease at increasing Ki-67, whereas for PODs such an OS gradient was evident only for Ki-67 less than about 55%, and the OS probability was very low for Ki-67 >55%. Considering G3 patients (Ki‑67 >20%) the above findings demonstrate that this group includes patients with different OS, according to Ki-67 values, and are in keeping with our previous results [7].

Abbreviations: OS, overall survival; WED, well-differentiated; POD, poorly-differentiated.


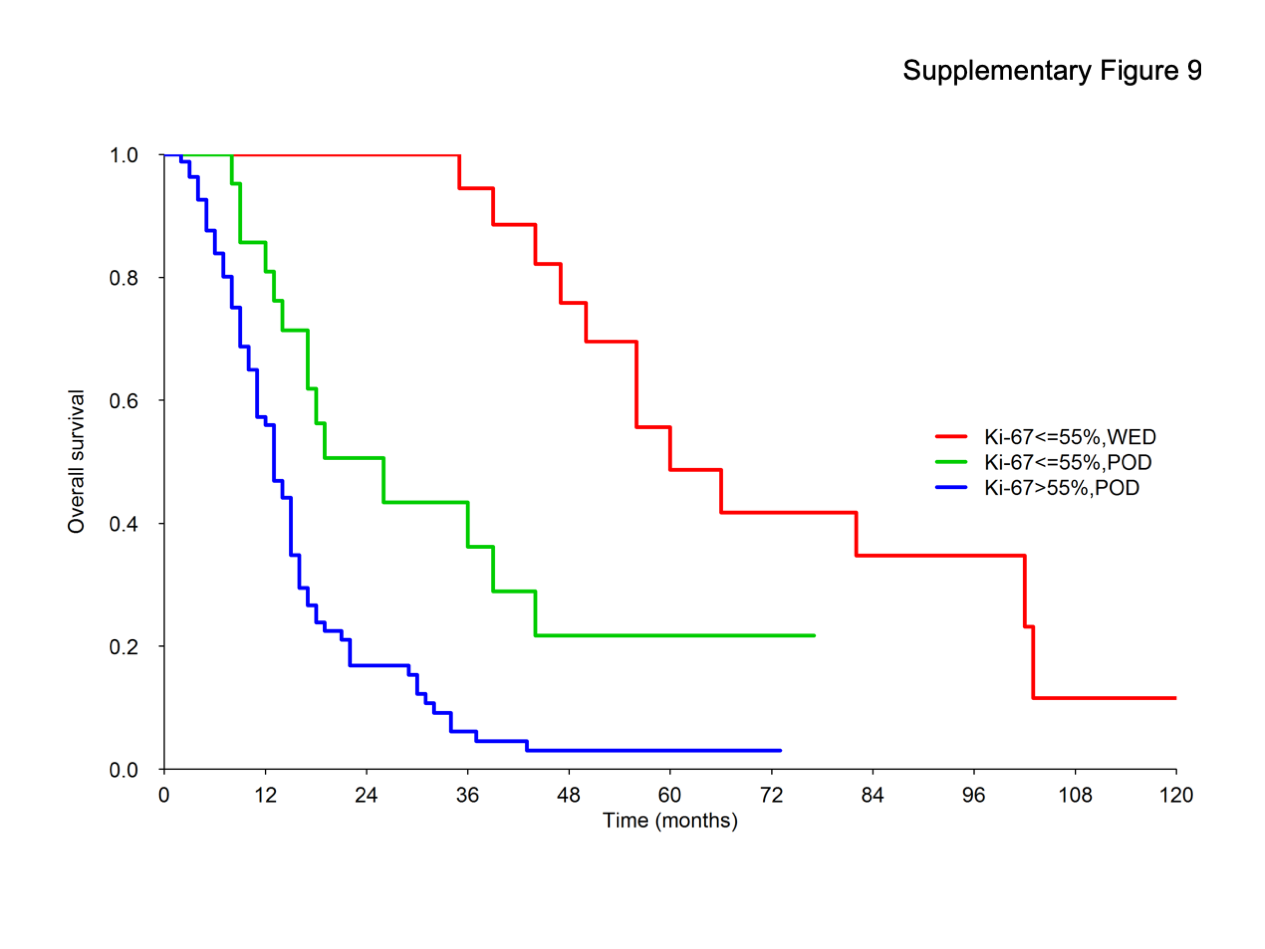


**Figure S9. Kaplan-Meier curves of OS in G3 patients according to Ki‑67 and morphology.**

Abbreviations: OS, overall survival; WED, well-differentiated; POD, poorly-differentiated.


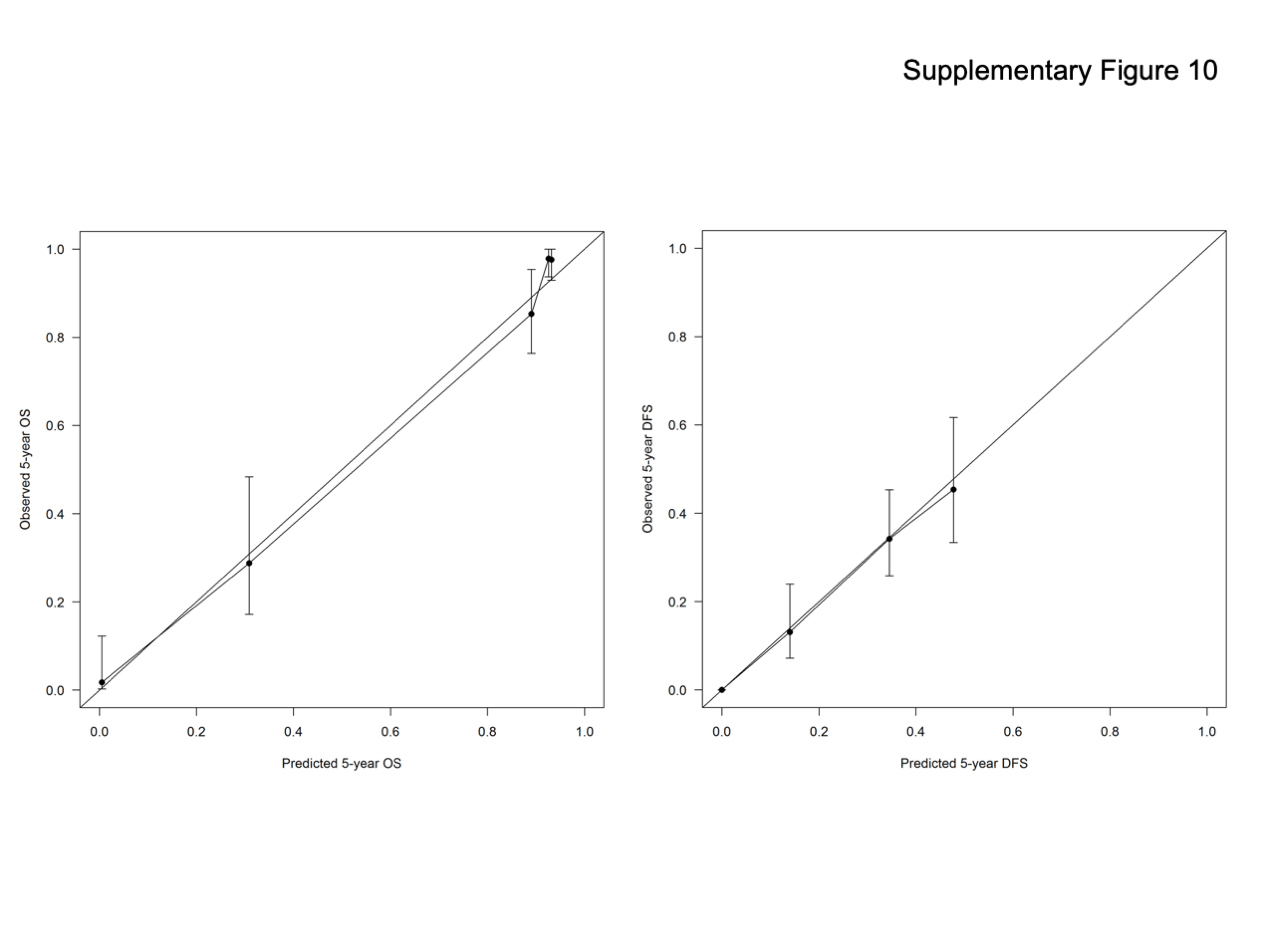


**Figure S10. Calibration plot for of the 5-year OS (left) and DFS (right) nomograms on the INT series.**

Explanation: the predicted OS/DFS probabilities were stratified into equally sized subgroups. For each subgroup, the average predicted probability (Predicted OS/DFS, x-axis) was plotted against the Kaplan Meier estimate (Observed OS/DFS, y-axis). The 95% confidence intervals of the Kaplan-Meier estimates are indicated with vertical lines. The dashed line indicates the reference line, which would indicate where an ideal nomogram would lie.

Abbreviations: OS, overall survival; DFS, disease-free survival; INT, Istituto Nazionale dei Tumori.


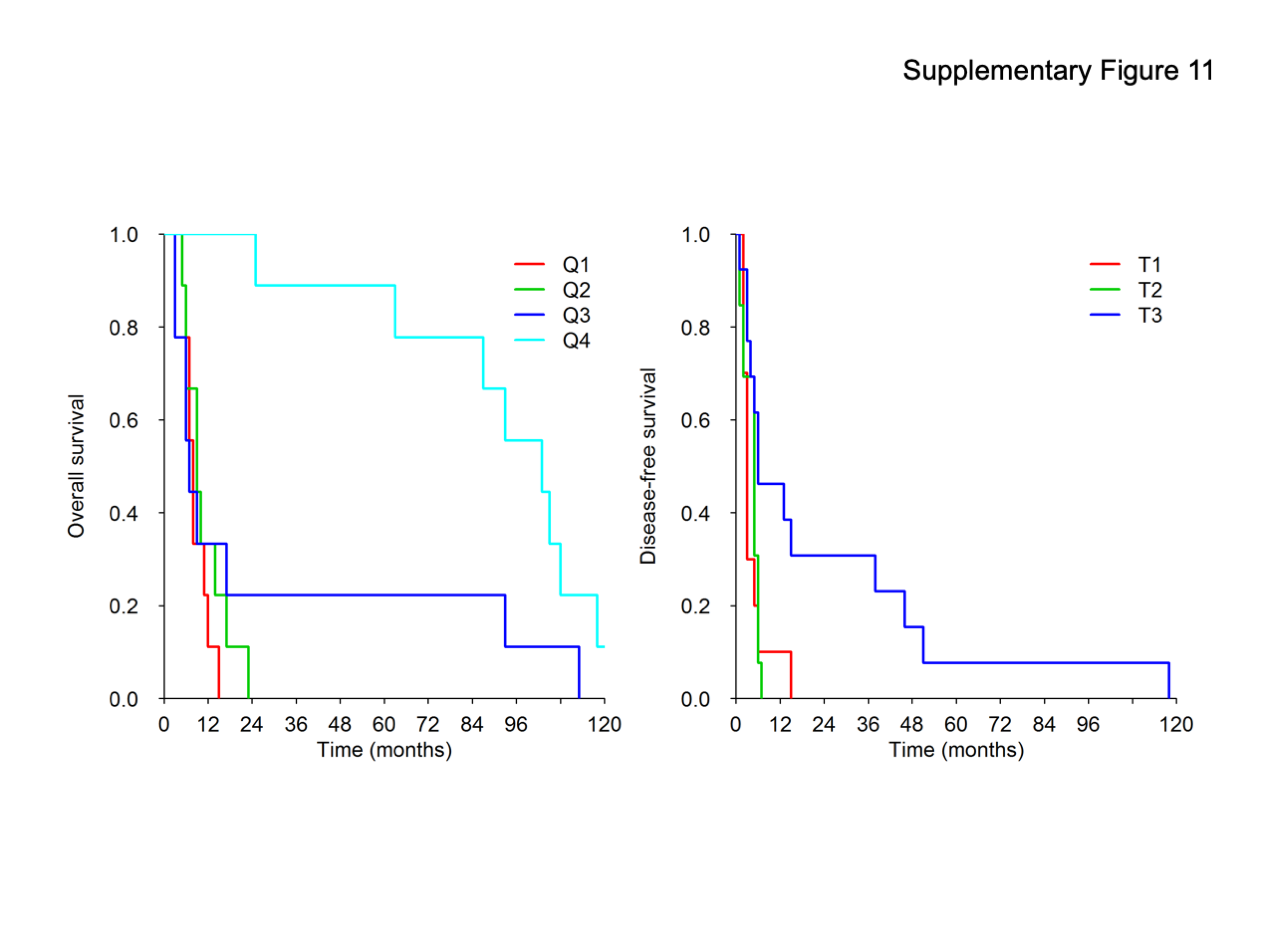
**Figure S11. Kaplan-Meier curves for OS (left) and DFS (right) according to the nomogram predictions on the HRH series.**

Left: the curves are stratified by quartiles of the nomogram-predicted 5-year OS probability. Right: the curves are stratified by tertiles of the nomogram-predicted 5-year DFS probability.

Abbreviations: OS, overall survival; DFS, disease-free survival; HRH, Humanitas Research Hospital.
