# Supplementary material for: Microenvironment and tumor inflammatory features improve prognostic prediction in gastro‐entero‐pancreatic neuroendocrine neoplasms
Source: J Pathol Clin Res. 2019 Jul 9;5(4):217–26. doi: 10.1002/cjp2.135 (PMC6817832; doi:10.1002/cjp2.135)
Supplement: Supplementary file 3 — Table S1. Antibody sources and dilutions Table S2. Univariable Cox model results for OS and DFS Table S3. Composition of MoTIFs PI for OS according to the selected variables COX‐2T, PD‐1S, CD8S, and HLA‐IS Table S4. Composition of MoTIFs PI for DFS according to the selected variables COX‐2T, PD‐1S, HLA‐IS, HLA‐IT, and HLA‐DRS [file CJP2-5-217-s003.docx]

**Microenvironment and tumor inflammatory features improve prognostic prediction in gastro-entero-pancreatic neuroendocrine neoplasms**

Milione M *et al. J Pathol Clin Res* DOI: 10.1002/cjp2.135

| **Table S1. Antibody sources and dilutions.** | | | | |
| --- | --- | --- | --- | --- |
| **Antigens** | **Dilution** | **Code Number** | **Clone** | **Source** |
| Ki-67 (M) | 1/400 | M7240 | Mib-1 | Dako, Agilent, Denmark |
| Synaptophysin (M) | 1/200 | M7315 | Dak-Synap | Dako, Agilent, Denmark |
| Chromogranin-A (M) | 1/100 | M0869 | Dak-A3 | Dako, Agilent, Denmark |
| CD3 (P) | 1/400 | A0452 | Polyclonal | Dako, Glostrup, Denmark |
| CD4 (M) | 1/300 | M7310 | 4B12 | Dako, Glostrup, Denmark |
| CD8 (M) | 1/20 | M7103 | C8/144B | Dako, Glostrup, Denmark |
| CD31 (M) | 1/200 | M0823 | JC/70 A | Dako, Glostrup, Denmark |
| α-SMA (M) | 1/800 | M0851 | 1A4 | Dako, Glostrup, Denmark |
| PD-L1 (M) | 1/50 | M3653 | 22C3 | Dako, Glostrup, Denmark |
| PD-1 (M) | 1/50 | ACI3137 | NAT105 | Biocare Medical |
| β-Catenin (M) | 1/1000 | 610154 | 14/Betacatenin | BD/Pharmingen San Diego,CA, USA |
| HLA-I (M) | 1/4000 | ab70328 | EMR8-5 | Abcam |
| HLA-DR (M) | 1/500 | MS-133-P0 | LN3 | Thermo fisher scientific |
| COX-2 (M) | 1/100 | 160112 | CX229 | Cayman Chemical Company |
| pS6 (phS6 Ser235/236) (M) | 1/200 | 4858 | D57.2.2E | Cell Signaling |
| NGFR (M) | 1/1000 | ab3125 | NGFR5 | Abcam |
| Abbreviations: M, monoclonal; P, polyclonal; Ki-67, Ki-67 index; α-SMA, Alpha-smooth muscle actin; PD-L1, Programmed death-ligand 1; PD-1, Programmed cell death-1; COX-2, Cyclooxygenase-2; pS6, Phospho-S6 Ribosomal Protein (Ser235/236); NGFR, Nerve growth factor receptor. | | | | |

| **Table S2. Univariable Cox model results for OS and DFS.** | | | | | | |
| --- | --- | --- | --- | --- | --- | --- |
|  | **OS** | | | **DFS** | | |
|  | **HR** | **95% CI** | ***p*** | **HR** | **95% CI** | ***p*** |
| HLA-I^T^* | 0.81 | 0.55-1.19 | <0.0001 | 0.64 | 0.49-0.84 | <0.0001 |
| CD3^S^ | 1.35 | 1.17-1.56 | <0.0001 | 1.04 | 0.86-1.25 | 0.001 |
| CD4^S^ | 1.13 | 1.02-1.26 | 0.017 | 1.12 | 1.04-1.21 | 0.005 |
| CD8^S^* | 0.59 | 0.39-0.89 | 0.003 | 0.66 | 0.48-0.89 | 0.007 |
| PD-1^S^ | 1.91 | 1.60-2.27 | <0.0001 | 1.59 | 1.38-1.84 | <0.0001 |
| PD-L1^S^* | 3.23 | 2.16-4.83 | <0.0001 | 2.10 | 1.55-2.86 | <0.0001 |
| HLA-I^S^* | 0.39 | 0.26-0.60 | <0.0001 | 0.51 | 0.38-0.68 | <0.0001 |
| HLA-DR^S^ | 0.48 | 0.34-0.68 | <0.0001 | 0.60 | 0.46-0.77 | <0.0001 |
| COX-2^T^ | 2.81 | 1.91-4.14 | <0.0001 | 1.95 | 1.57-2.43 | <0.0001 |
| pS6^T^ | 1.12 | 0.94-1.33 | 0.195 | 1.07 | 0.94-1.21 | 0.297 |
| β-catenin^s/c-T^ * | 5.50 | 3.73-8.12 | <0.0001 | 2.42 | 1.89-3.10 | <0.0001 |
| β-catenin^n-T^.c |  |  | <0.0001 |  |  | <0.0001 |
| Absent *vs* Abs all | 0.99 | 0.40-2.46 |  | 0.85 | 0.49-1.50 |  |
| Present *vs* Abs all | 10.26 | 4.01-26.26 |  | 4.32 | 2.36-7.90 |  |
| NGFR^S^* | 0.41 | 0.27-0.60 | <0.0001 | 0.69 | 0.52-0.91 | 0.027 |
| COX-2^S^* | 0.71 | 0.48-1.06 | <0.0001 | 0.84 | 0.64-1.11 | 0.028 |
| pS6^S^* | 0.35 | 0.22-0.55 | <0.0001 | 0.62 | 0.45-0.84 | 0.003 |
| Morphology |  |  | <0.0001 |  |  | <0.0001 |
| POD *vs* WED | 30.49 | 18.59-49.68 |  | 8.79 | 6.45-11.97 |  |
| Ki-67* | 31.42 | 18.77-52.61 | <0.0001 | 5.53 | 4.26-7.17 | <0.0001 |

Abbreviations: OS, overall survival; DFS, disease-free survival; HR, hazard ratio; CI, confidence interval; *p*, p value at the Wald test, testing the global association between OS/DFS and each variable; thus for the numerical features the p value is not referred to the specific comparison between the 3^rd^ and 1^st^ quartiles; POD, poorly-differentiated; WED, well-differentiated; Abs all, absent at all sites.

For the numerical features the HRs were estimated to compare the third versus the first quartile of the variable distribution.

* The nonlinear term of the 3-knots restricted cubic spline was statistically significant [5].

**Table S3. Composition of MoTIFs PI for OS according to the selected variables COX-2^T^, PD-1^S^, CD8^S^, and HLA-I^S^.**

| Feature  scores | **PI values** | | | | | | | | | |
| --- | --- | --- | --- | --- | --- | --- | --- | --- | --- | --- |
|  | **0** | | **1** | | **2** | | **3** | | **4** | |
|  | n=32 | 10.2% | n=121 | 38.5% | n=96 | 30.6% | n=48 | 15.3% | n=17 | 5.4% |
| 0 0 0 0 | 32 | 100.0% |  |  |  |  |  |  |  |  |
| 1 0 0 0 |  |  | 55 | 45.5% |  |  |  |  |  |  |
| 0 1 0 0 |  |  | 6 | 5.0% |  |  |  |  |  |  |
| 0 0 1 0 |  |  | 43 | 35.5% |  |  |  |  |  |  |
| 0 0 0 1 |  |  | 17 | 14.0% |  |  |  |  |  |  |
| 1 1 0 0 |  |  |  |  | 50 | 52.1% |  |  |  |  |
| 1 0 1 0 |  |  |  |  | 15 | 15.6% |  |  |  |  |
| 1 0 0 1 |  |  |  |  | 20 | 20.8% |  |  |  |  |
| 0 1 1 0 |  |  |  |  | --- | --- |  |  |  |  |
| 0 1 0 1 |  |  |  |  | 9 | 9.4% |  |  |  |  |
| 0 0 1 1 |  |  |  |  | 2 | 2.1% |  |  |  |  |
| 1 1 1 0 |  |  |  |  |  |  | 9 | 18.8% |  |  |
| 1 1 0 1 |  |  |  |  |  |  | 12 | 25.0% |  |  |
| 1 0 1 1 |  |  |  |  |  |  | 26 | 54.2% |  |  |
| 0 1 1 1 |  |  |  |  |  |  | 1 | 2.1% |  |  |
| 1 1 1 1 |  |  |  |  |  |  |  |  | 17 | 100.0% |

NOTE. The figures in the first column represent the observed combination of the feature scores; positivity (score=1) was as follows: COX-2^T^>4, PD-1^S^>0, CD8^S^<1, and HLA-I^S^<1. The PI is equal to the sum of the 4 scores. Among the 121 patients with PI=1, only a few had either positive PD-1^S^ (5%) or HLA-I^S^ (14%); 52.1% of the 96 PI=2 patients had positive COX-2^T^ coupled with positive PD-1^S^, and 36.4% had positive COX-2^T^ paired with positive CD8^S^ and HLA-I^S^; among the 48 PI=3 patients, 54.2% had positive COX-2^T^, CD8^S^ and HLA-I^S^ (see also Supplementary Figure 5 left). Abbreviations: MoTIFs, microenvironment and tumor inflammatory features; PI, prognostic index; OS, overall survival; n, number of patients.

**Table S4. Composition of MoTIFs PI for DFS according to the selected variables COX-2^T^, PD-1^S^, HLA-I^S^, HLA-I^T^, and HLA-DR^S^.**

| Feature scores | **PI values** | | | | | | | | | | | |
| --- | --- | --- | --- | --- | --- | --- | --- | --- | --- | --- | --- | --- |
|  | **0** | | **1** | | **2** | | **3** | | **4** | | **5** | |
|  | n=53 | 16.9% | n=122 | 38.9% | n=77 | 24.5% | n=31 | 9.9% | n=29 | 9.2% | n=2 | 0.6% |
| 0 0 0 0 0 | 53 | 100.0% |  |  |  |  |  |  |  |  |  |  |
| 1 0 0 0 0 |  |  | 74 | 60.7% |  |  |  |  |  |  |  |  |
| 0 1 0 0 0 |  |  | 3 | 2.5% |  |  |  |  |  |  |  |  |
| 0 0 1 0 0 |  |  | 21 | 17.2% |  |  |  |  |  |  |  |  |
| 0 0 0 1 0 |  |  | --- | --- |  |  |  |  |  |  |  |  |
| 0 0 0 0 1 |  |  | 24 | 19.7% |  |  |  |  |  |  |  |  |
| 1 1 0 0 0 |  |  |  |  | 22 | 28.6% |  |  |  |  |  |  |
| 1 0 1 0 0 |  |  |  |  | 24 | 31.2% |  |  |  |  |  |  |
| 1 0 0 1 0 |  |  |  |  | 12 | 15.6% |  |  |  |  |  |  |
| 1 0 0 0 1 |  |  |  |  | 13 | 16.9% |  |  |  |  |  |  |
| 0 1 1 0 0 |  |  |  |  | 1 | 1.3% |  |  |  |  |  |  |
| 0 1 0 1 0 |  |  |  |  | --- | --- |  |  |  |  |  |  |
| 0 1 0 0 1 |  |  |  |  | --- | --- |  |  |  |  |  |  |
| 0 0 1 1 0 |  |  |  |  | 4 | 5.2% |  |  |  |  |  |  |
| 0 0 1 0 1 |  |  |  |  | --- | --- |  |  |  |  |  |  |
| 0 0 0 1 1 |  |  |  |  | 1 | 1.3% |  |  |  |  |  |  |
| 1 1 1 0 0 |  |  |  |  |  |  | 3 | 9.7% |  |  |  |  |
| 1 1 0 1 0 |  |  |  |  |  |  | --- | --- |  |  |  |  |
| 1 1 0 0 1 |  |  |  |  |  |  | 3 | 9.7% |  |  |  |  |
| 1 0 1 1 0 |  |  |  |  |  |  | 11 | 35.5% |  |  |  |  |
| 1 0 1 0 1 |  |  |  |  |  |  | 6 | 19.4% |  |  |  |  |
| 1 0 0 1 1 |  |  |  |  |  |  | 5 | 16.1% |  |  |  |  |
| 0 1 1 1 0 |  |  |  |  |  |  | --- | --- |  |  |  |  |
| 0 1 1 0 1 |  |  |  |  |  |  | 2 | 6.5% |  |  |  |  |
| 0 0 1 1 1 |  |  |  |  |  |  | 1 | 3.2% |  |  |  |  |
| 1 1 1 1 0 |  |  |  |  |  |  |  |  | 4 | 13.8% |  |  |
| 1 1 1 0 1 |  |  |  |  |  |  |  |  | 2 | 6.9% |  |  |
| 1 1 0 1 1 |  |  |  |  |  |  |  |  | --- | --- |  |  |
| 0 1 1 1 1 |  |  |  |  |  |  |  |  | --- | --- |  |  |
| 1 0 1 1 1 |  |  |  |  |  |  |  |  | 23 | 79.3% |  |  |
| 1 1 1 1 1 |  |  |  |  |  |  |  |  |  |  | 2 | 100.0% |

NOTE. The figures in the first column represent the observed combination of the feature scores; positivity (score = 1) was as follows: COX-2^T^>4, PD-1^S^>4, HLA-I^S^<1, HLA-I^T^<2, HLA-DR^S^<6. The PI is equal to the sum of the 5 scores. Among the 122 patients with PI=1, only a few had positive PD-1^S^ (2.5%), none HLA-I^T^ and 60.7% COX-2^T^; 59.8% of the 77 PI=2 patients had positive COX-2^T^ paired with positive PD-1^S^ or HLA-I^S^, and 32.5% had positive COX-2^T^ paired with positive HLA-I^S^ or HLA-DR^S^; among the 31 PI=3 patients, 35.5% had positive COX-2^T^, HLA-I^S^ and HLA-I^T^, and 35.5% had positive COX-2^T^ and HLA-DR^S^ associated with HLA-I^S^ or HLA-I^T^; 79.3% of 29 PI=4 patients had negative only PD-1^S^ (see also Figure S5 right). Abbreviations: MoTIFs, microenvironment and tumor inflammatory features; PI, prognostic index; DFS, disease-free survival; n, number of patients.
